# Cerium oxide and barium sulfate nanoparticle inhalation affects gene expression in alveolar epithelial cells type II

**DOI:** 10.1186/s12951-018-0343-4

**Published:** 2018-02-20

**Authors:** Daniela Schwotzer, Monika Niehof, Dirk Schaudien, Heiko Kock, Tanja Hansen, Clemens Dasenbrock, Otto Creutzenberg

**Affiliations:** Fraunhofer Institute for Toxicology and Experimental Medicine ITEM, Nikolai-Fuchs-Straße 1, 30625 Hannover, Germany

**Keywords:** Nanoparticles, Gene expression, CeO_2_, BaSO_4_, Inhalation, Biomarkers, Alveolar epithelial cells type II, Inflammation, Oxidative stress

## Abstract

**Background:**

Understanding the molecular mechanisms of nanomaterial interacting with cellular systems is important for appropriate risk assessment. The identification of early biomarkers for potential (sub-)chronic effects of nanoparticles provides a promising approach towards cost-intensive and animal consuming long-term studies. As part of a 90-day inhalation toxicity study with CeO_2_ NM-212 and BaSO_4_ NM-220 the present investigations on gene expression and immunohistochemistry should reveal details on underlying mechanisms of pulmonary effects. The role of alveolar epithelial cells type II (AEII cells) is focused since its contribution to defense against inhaled particles and potentially resulting adverse effects is assumed. Low dose levels should help to specify particle-related events, including inflammation and oxidative stress.

**Results:**

Rats were exposed to clean air, 0.1, 0.3, 1.0, and 3.0 mg/m^3^ CeO_2_ NM-212 or 50.0 mg/m^3^ BaSO_4_ NM-220 and the expression of 391 genes was analyzed in AEII cells after one, 28 and 90 days exposure. A total number of 34 genes was regulated, most of them related to inflammatory mediators. Marked changes in gene expression were measured for Ccl2, Ccl7, Ccl17, Ccl22, Ccl3, Ccl4, Il-1α, Il-1ß, and Il-1rn (inflammation), Lpo and Noxo1 (oxidative stress), and Mmp12 (inflammation/lung cancer). Genes related to genotoxicity and apoptosis did not display marked regulation. Although gene expression was less affected by BaSO_4_ compared to CeO_2_ the gene pattern showed great overlap. Gene expression was further analyzed in liver and kidney tissue showing inflammatory responses in both organs and marked downregulation of oxidative stress related genes in the kidney. Increases in the amount of Ce were measured in liver but not in kidney tissue. Investigation of selected genes on protein level revealed increased Ccl2 in bronchoalveolar lavage of exposed animals and increased Lpo and Mmp12 in the alveolar epithelia.

**Conclusion:**

AEII cells contribute to CeO_2_ nanoparticle caused inflammatory and oxidative stress reactions in the respiratory tract by the release of related mediators. Effects of BaSO_4_ exposure are low. However, overlap between both substances were detected and support identification of potential early biomarkers for nanoparticle effects on the respiratory system. Signs for long-term effects need to be further evaluated by comparison to a respective exposure setting.

**Electronic supplementary material:**

The online version of this article (10.1186/s12951-018-0343-4) contains supplementary material, which is available to authorized users.

## Background

Nanotechnologies and -materials are of great interest for product development and since several years its application is getting more popular. For safe use by manufacturers and consumers assessment of toxicity and adverse health effects is required. The present investigations are part of a comprehensive project on the toxicity and carcinogenicity of nanoparticles with nano-CeO_2_ as representative material in different concentrations covering the low to moderate dose range (0.1, 0.3, 1.0, 3.0 mg/m^3^), and nano-BaSO_4_ at one high concentration (50.0 mg/m^3^) for comparison to a non-toxic and safe material. The project is funded by the German Federal Ministry of Education and Research (03X0149) and should provide supporting data on a combined chronic inhalation toxicity and carcinogenicity study with cerium oxide and barium sulfate nanoparticles [NANoREG (81|0661/10|170)]. The automotive industry uses CeO_2_ nanoparticles in catalyzers and as fuel additive because of the material’s catalytic properties. Due to its chemical inertness, barium sulfate is used in multiple applications e.g. as filler in the biomedical sector. The nanoparticles were examined in a 90-day inhalation toxicity study with obligatory endpoints according to OECD TG 413 [1]. To identify early biomarkers for long-term effects of nanoparticles and investigate potential mechanisms of action, highly sensitive methods (gene expression analysis, immunohistochemistry) were further included in the project and are described in the present article.

Depending on their size and the striving to form agglomerates, nanoparticles penetrate different parts of the respiratory tract and likely end up in the alveolar space. They potentially react with cellular systems and induce inflammatory reactions or other molecular events. They further might pass the air–blood-barrier and translocate to extra-pulmonary organs. One major mechanism for the elimination of particles from the respiratory tract is the uptake by alveolar macrophages and clearance via the mucociliary escalator or the lymphatic system. However, the alveolar space consists of further cell types involved in host defense and lung function maintenance. Alveolar epithelial type II (AEII) cells are responsible for production and recycling of lung surfactant, play a role in turnover of the alveolar epithelia, and bear the ability to transform into alveolar epithelial type I (AEI) cells (e.g. for replacement of damaged cells). It has early been reviewed that AEII cells are an important component of the respiratory defense system against foreign material, including nanoparticles [2]. They produce and secrete a variety of factors, like surfactant proteins and chemokines to recruit macrophages and induce inflammatory processes for substance elimination [2–6]. It has been shown that carbon black nanoparticles, but not fine or nano-TiO_2_, or fine carbon black stimulate AEII cells to release factors for macrophage migration in vitro [7]. Furthermore, AEII cells and not macrophages are assumed to be the main producers of neutrophil attracting chemokines in the early inflammatory response to carbon nanoparticles administered via intratracheal instillation [8]. AEII cells might also internalize nanomaterial via different mechanisms, including the route of surfactant recycling [9–13]. In vivo studies on gold nanoparticles demonstrate the presence of the respective nanomaterial in lamellar bodies of AEII cells or the lung lining fluid [14, 15].

The presence of nanomaterial in the lung can cause chronic inflammatory reactions of lung tissue (e.g. after repeated administration), which can eventually result into long-term adverse effects like fibrosis or neoplastic lesions. It has recently been demonstrated that AEII cells play a role in CeO_2_ nanoparticle induced lung fibrosis [16]. Furthermore, it is known that AEII cells are a potential progenitor for lung tumors. As reviewed by Oberdörster [17] during particle-related inflammation, the recruited immune cells release growth factors and induce oxidative stress by the production of reactive oxygen species (ROS) for host defense. ROS can cause DNA damage, growth factors stimulate cell proliferation and by this the risk of tumor formation is increased. This hypothesis includes the well investigated process of secondary genotoxicity which is often accompanied by a present overload situation [18]. In contrast, little is known about primary mechanisms of genotoxicity, which are attributed to direct particle effects, like ROS generation due to surface reactivity [18]. Investigations of respective effects need to be done at absent inflammation. Less knowledge is published in how far CeO_2_ nanoparticles react via the described molecular mechanisms and if they bear a carcinogenic potential after inhalation. Several in vivo inhalation studies demonstrate the induction of inflammatory reactions [19–26]. However, the majority of these studies most likely describe effects occurring during a present overload situation. Pro-oxidative but also anti-oxidative effects have been demonstrated for CeO_2_ nanoparticles [19, 27–32]. Furthermore, some research indicates a genotoxic potential in vivo (intratracheal or oral application) [30, 33, 34] or in vitro [29, 35]. In contrast, first results from the chronic study mentioned earlier as well as the related dose-range finding study indicate no genotoxic effects for CeO_2_ [22, 36]. Investigations of Ma et al. [16, 37, 38] indicate fibrosis induction after CeO_2_ intratracheal instillation. More mechanistic information is needed for better understanding of partially contrary hypotheses. This includes the contribution of inflammation and overload on the one hand, and the particle’s reactivity on the other hand, to the respective molecular events. Also, the use of very high nanoparticle concentrations in most available studies requires more research on lower dose levels.

Since the major share of the inhaled nanoparticle dose usually deposits in the respiratory organs and is eliminated via mechanisms of pulmonary clearance, extra-pulmonary translocation of a smaller particle fraction is a frequently detected concomitant effect. Aalapati et al. [19] measured Ce contents in different extra-pulmonary organs after 28-day exposure in mice, with highest levels in liver and kidney and particle-related histopathological changes of the respective organs. Other in vivo studies display comparable effects for CeO_2_ nanoparticles [30, 39–43].

Potential mechanisms of action, especially regarding carcinogenicity of CeO_2_ and BaSO_4_ nanoparticles, were examined in this study. The highly sensitive method of gene expression analysis aims on the identification of early biomarkers for nanoparticle-related effects, especially those occurring after long-term exposure to realistic, low substance levels. The inclusion of low CeO_2_ nanoparticle concentrations should create a situation of absent overload and inflammation to identify substance-related pro-oxidative, pro-proliferative or apoptotic as well as genotoxic effects. We focused on AEII cells as they are potential key players in (particle induced) pulmonary toxicity. The contribution of extra-pulmonary translocation to nanoparticle toxicity mechanisms motivated additional examinations of liver and kidney tissue as organs responsible for substance elimination. The identification of early biomarkers after acute to subchronic nanoparticle exposure creates a step towards the reduction of cost-intensive, animal-consuming long-term in vivo studies. Moreover, the in vivo markers should serve as basis for further investigations in an in vitro nanoparticle-exposure setup to intensify the focus on alternatives to animal testing.

## Results

### Conventional endpoints according to OECD TG 413

The obligatory investigations of the 90-day inhalation toxicity study according to OECD TG 413 were comprehensively reported elsewhere [1] and are briefly summarized in Table 1. We detected increasing lung burden levels for both nanomaterials with reduced clearance halftimes of cerium at concentrations of ≥ 1.0 mg/m^3^. Contrastingly, barium was rapidly cleared from the lung. The response of lung tissue and associated cell populations was dominated by inflammatory reactions. Particle-laden macrophages and inflammatory cell infiltrations, directed by neutrophils were detected in histopathology and bronchoalveolar lavage (BAL) analysis. Immunohistochemistry markers for genotoxicity (CeO_2_ only) and cell proliferation were increased with ongoing exposure. Effects remained persistent up to 90 days subsequent to CeO_2_, and although to a much lower extent also after BaSO_4_ exposure.

**Table 1:** Summary of conventional endpoints

Investigation	CeO_2_	BaSO_4_
Retention analytics	↑ lung burden (time- and concentration-dependent); ↓ clearance (≥ 1.0 mg/m^3^)	↑ lung burden (time-dependent); rapid clearance
Hematology/clinical chemistry (d90 + 1rec)	↑ blood neutrophils	NAD
BAL analysis	↑ neutrophils, lymphocytes, total protein, LDH, β-glucuronidase (time-/concentration-dependent); post-exposure persistency	↑ neutrophils; no post-exposure persistency
Histopathology	↑ particle-laden macrophages and inflammatory cell infiltrations (alveolar/interstitial/lymphoid tissue), bronchiolo-alveolar hyperplasia and fibrosis; post-exposure persistency	↑ particle-laden macrophages and inflammatory cell infiltrations; intra-epithelial eosinophilic globules and mucus cell hyperplasia in the nasal cavity; partly post-exposure persistency
Immunohistochemistry	↑ γ-H2AX, 8-OHdG (genotoxicity); ↑ Ki-67 (proliferation); post-exposure persistency	↑ Ki-67 (proliferation); partly post-exposure persistency

*NAD* no abnormalities detected

### Gene expression analysis in AEII cells

**Fig. 1:**
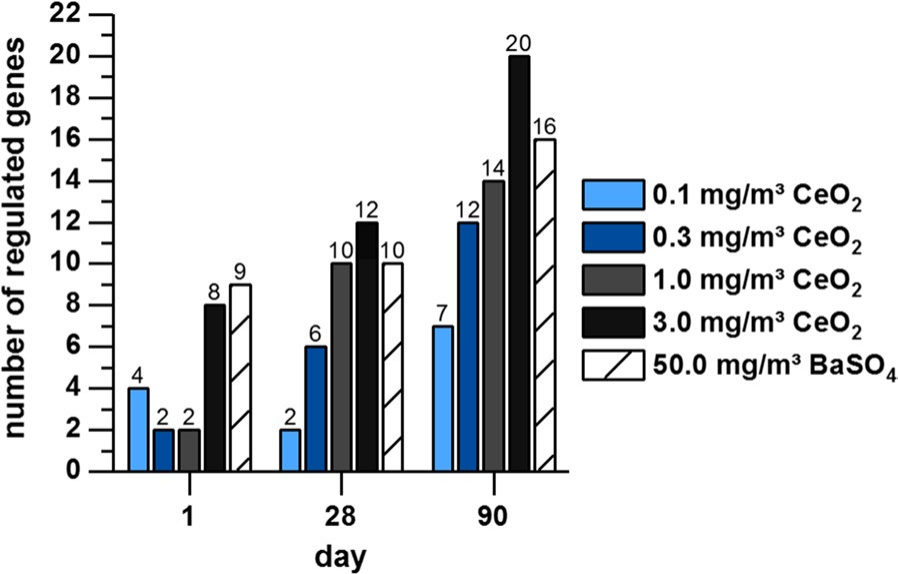
Number of regulated genes per concentration and time point. The number of regulated genes in AEII cells isolated from rats exposed to 0.1, 0.3, 1.0 or 3.0 mg/m^3^ CeO_2_ or 50.0 mg/m^3^ BaSO_4_ nanoparticles for one, 28 or 90 days is illustrated

**Fig. 2:**
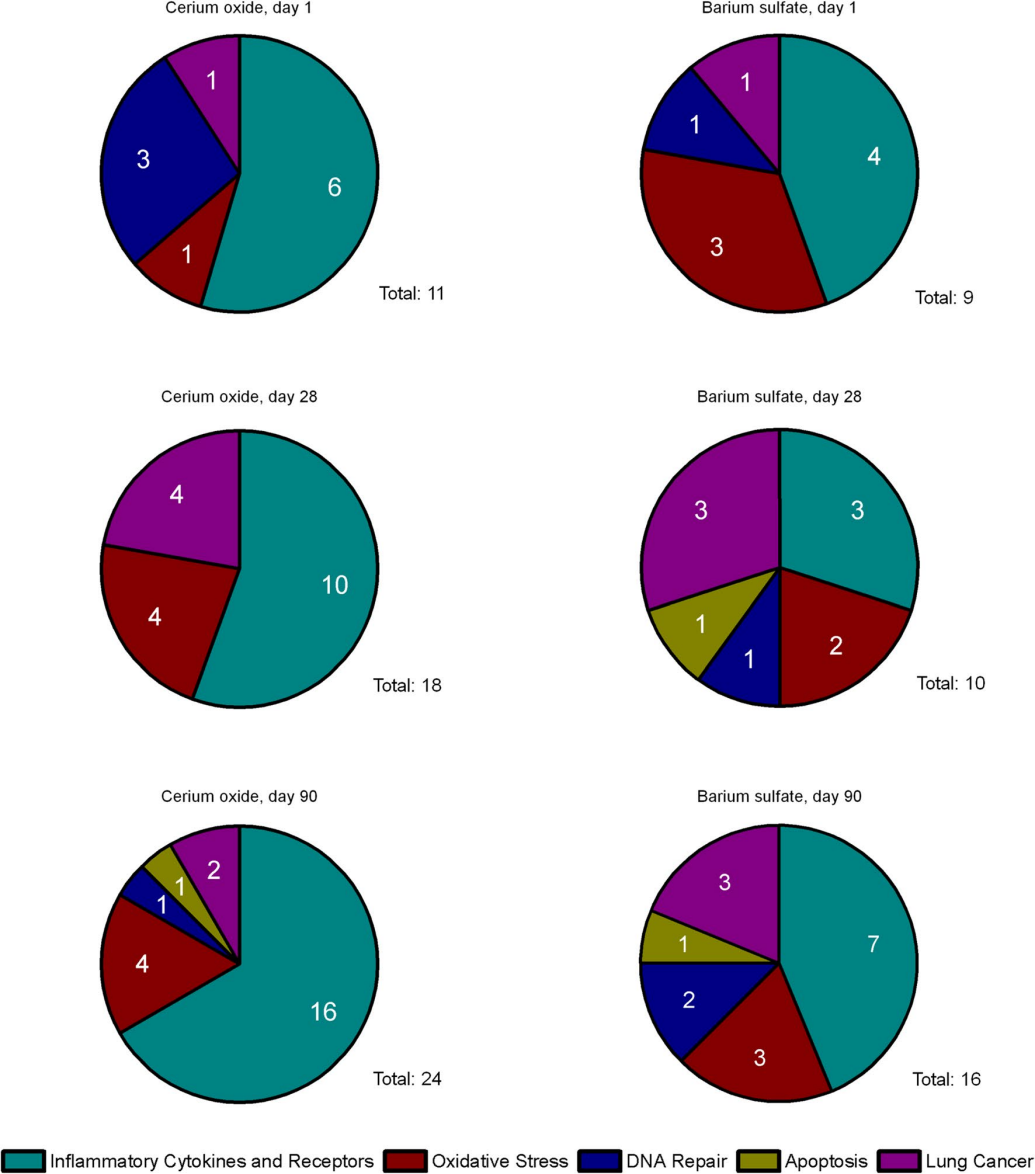
Distribution of regulated genes over the analyzed pathways. The number of regulated genes in isolated AEII cells after CeO_2_ nanoparticle (pool of all dose groups) and BaSO_4_ nanoparticle exposure is illustrated per time point and pathway

For AEII cells five different profiler PCR arrays (inflammatory cytokines and receptors, oxidative stress, DNA repair, apoptosis, lung cancer) were analyzed. In total, 34 genes were regulated and are listed in Table 2 and Additional file 1: Table S1. An overall upregulation of gene expression has been observed rather than downregulation. Switches between up- and downregulation of one specific gene has been detected rarely, therefore the direction of gene regulation could be determined quite explicit for most of the genes. Furthermore, the overlap between the regulated genes in response to both substances was quite high (> 60% similarity). The number of regulated genes increased with ongoing exposure time and increasing CeO_2_ concentration (Fig. 1). Analysis of gene distribution over the different pathways revealed major contribution of inflammatory mediators at all time points (Fig. 2). All other pathways yielded up to four regulated genes. Inflammatory- and oxidative stress-related gene numbers increased in response to CeO_2_. Also in response to BaSO_4_ a time-dependent increase of the number of regulated genes was detected. However, the distribution over the five specified groups of mediators did not increase significantly. In total, 30 different genes were affected by CeO_2_ and 25 genes by BaSO_4_ exposure. The difference was mainly due to a lower number of inflammatory mediators regulated by BaSO_4_. Data for the most promising genes regarding their function as potential marker for nanoparticle-related effects and linked mechanisms of action are displayed individually in the following sections. Figure 15 shows an overview of assumed mechanistic relationships between the different cell types and effects involved in CeO_2_ nanoparticle responses.

**Table 2:** Gene regulation in AEII cells in response to CeO_2_ and BaSO_4_ after different exposure periods

Array	Gene		CeO_2_			BaSO_4_		
			1 day	28 days	90 days	1 day	28 days	90 days
Inflammatory cytokines and receptors	Ccl3	C–C motif chemokine ligand 3						
	Ccl17	C–C motif chemokine ligand 17						
	Cx3cr1	C–X3–C motif chemokine receptor 1						
	Il1α	Interleukin 1 alpha						
	Il1β	Interleukin 1 beta						
	Il1rn	Interleukin 1 receptor antagonist						
	Tnfsf4	Tumor necrosis factor superfamily member 4						
	Ccl2	C–C motif chemokine ligand 2						
	Ccl4	C–C motif chemokine ligand 4						
	Ccl7	C–C motif chemokine ligand 7						
	Ccl11	C–C motif chemokine ligand 11						
	Ccl20	C–C motif chemokine ligand 20						
	Ccl22	C–C motif chemokine ligand 22						
	Ccl24	C–C motif chemokine ligand 24						
	Pf4	platelet factor 4						
	Cxcl9	C–X–C motif chemokine ligand 9						
	Cd40lg	CD40 ligand						
	Il2rb	Interleukin 2 receptor, beta						
Oxidative stress	Lpo	Lactoperoxidase						
	Noxo1	NADPH oxidase organizer 1						
	Hba1	Hemoglobin alpha, adult chain 2						
	Scd1	Stearoyl-Coenzyme A desaturase 1						
	Krt1	Keratin 1						
DNA repair	Msh5	mutS homolog 5						
	Exo1	Exonuclease 1						
	Mutyh	MutY homolog (*E. coli*)						
Apoptosis	Birc5	Baculoviral IAP repeat-containing 5						
Lung cancer	Cyp1b1	Cytochrome P450, family 1, subfamily b, polypeptide 1						
	Fabp4	Fatty acid binding protein 4						
	Mmp12	Matrix metallopeptidase 12						
	Opcml	Opioid binding protein/cell adhesion molecule-like						
	Tcf21	Transcription factor 21						
	Thbs2	Thrombospondin 2						
	Top2a	Topoisomerase (DNA) II alpha						
Total	34		11	18	24	9	10	15

upregulation;  downregulation;  up- or downregulation (differences between CeO_2_ dose groups); cut-off: FR ≤ − 2.0 or ≥ 2.0

#### Inflammatory cytokines and receptors

**Fig. 3:**
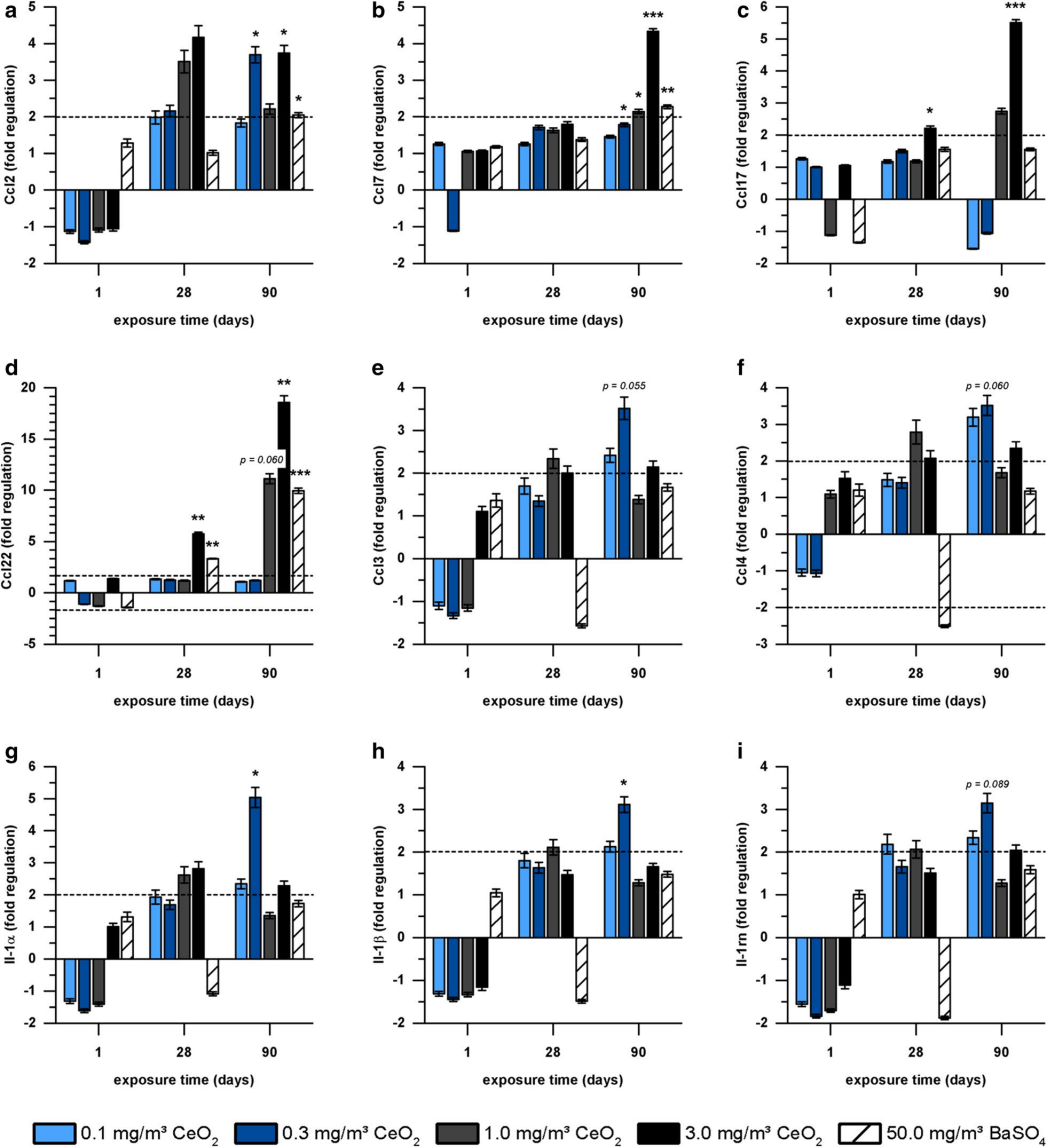
Gene expression of inflammatory mediators in AEII cells. Gene expression of **a** Ccl2, **b** Ccl7, **c** Ccl17, **d** Ccl22, **e** Ccl3, **f** Ccl4, **g** Il-1α, **h** Il-1ß, and **i** Il-1rn in AEII cells of rats exposed to 0.1, 0.3, 1.0 or 3.0 mg/m^3^ CeO_2_ or 50.0 mg/m^3^ BaSO_4_ nanoparticles for one, 28 or 90 days is illustrated. Values are expressed as mean fold regulation of clean air control ± SD; cut-off: fold regulation ≤ − 2.0 or ≥ 2.0 (dotted line), *p < 0.05, **p < 0.01, ***p < 0.001; n ≤ 5; Student’s T-test analysis of the replicate 2^−∆Ct^ values for each gene in the control and treatment groups

The chemokines Ccl2, Ccl7, Ccl17 and Ccl22 (Fig. 3a–d) showed the most distinct upregulation of all inflammatory mediators tested. Regulation was highest after 90 days exposure to 3.0 mg/m^3^ CeO_2_. Especially Ccl22 (Fig. 3d) showed a very high response to CeO_2_ with a gene expression up to 19-fold higher than the control group. The regulation pattern for these four chemokines is quite similar. The inflammatory mediators Ccl3, Ccl4, Ccl24, Il-1α, Il-1β, Il-1rn were upregulated at low CeO_2_ concentrations (0.1, 0.3 mg/m^3^) with a similar expression pattern (Fig. 3e–i). Although slight upregulation was detected for several mediators after 28 days, effects were highest after 90 days exposure. In contrast, BaSO_4_ only affected Ccl2, Ccl7, and Ccl22.

#### Oxidative stress

**Fig. 4:**
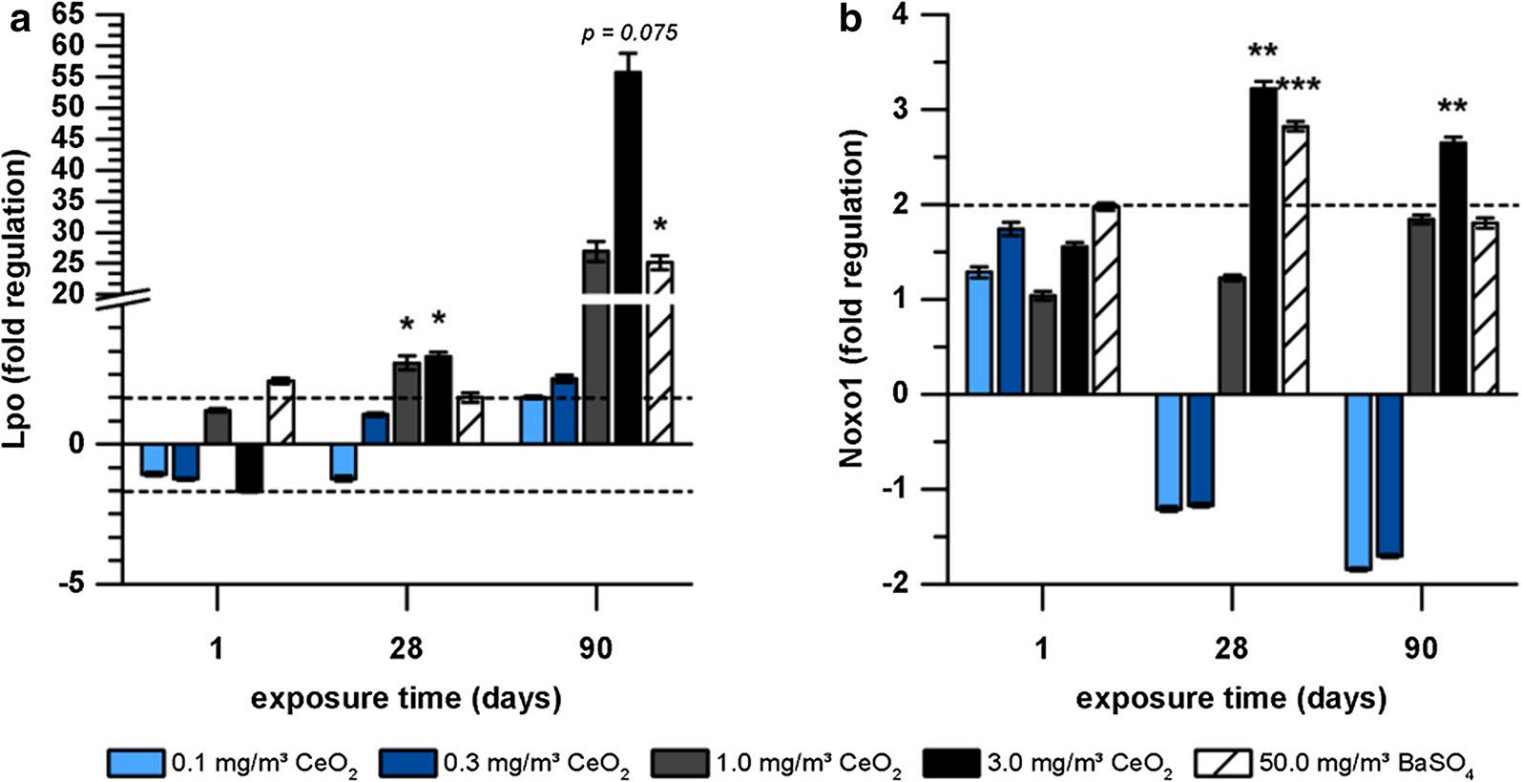
Expression of oxidative stress-related genes in AEII cells in response to nanoparticle exposure. Gene expression of **a** Lpo and **b** Noxo1 in AEII cells of rats exposed to 0.1, 0.3, 1.0 or 3.0 mg/m^3^ CeO_2_ or 50.0 mg/m^3^ BaSO_4_ nanoparticles for one, 28 or 90 days is illustrated. Values are expressed as mean fold regulation of clean air control ± SD; cut-off: fold regulation ≤ − 2.0 or ≥ 2.0 (dotted line), *p < 0.05, **p < 0.01, ***p < 0.001; n ≤ 5; Student’s T-test analysis of the replicate 2^−∆Ct^ values for each gene in the control and treatment groups

Four genes related to oxidative stress were regulated after CeO_2_ nanoparticle exposure (Table 2). The highest responses were detected for Lpo and Noxo1 with a concentration- and time-dependent increase (Fig. 4). Expression levels exceeded the fold regulation cut off already at 0.3 mg/m^3^ CeO_2_ nanoparticle exposure and reached values up to 56-fold higher compared to clean air inhalation after 90 days exposure to 3.0 mg/m^3^ CeO_2_. Noxo1 was significantly upregulated after 28 and 90 days in response to the highest concentration of CeO_2_. BaSO_4_ exposure revealed a total number of five regulated genes related to oxidative stress (Table 2). Lpo was affected in a similar way as after CeO_2_ exposure, with a peak fold regulation of 25. Noxo1 was upregulated only after 28 days exposure to BaSO_4_.

#### DNA repair and apoptosis

Three genes related to DNA repair were regulated (Table 2), whereas Exo1 showed the most distinct regulation (data not shown). The impact of BaSO_4_ on Exo1 expression was higher than effects of CeO_2_. Birc5, an apoptosis-related gene was slightly upregulated after 90-day nanoparticle exposure (data not shown).

#### Lung cancer

**Fig. 5:**
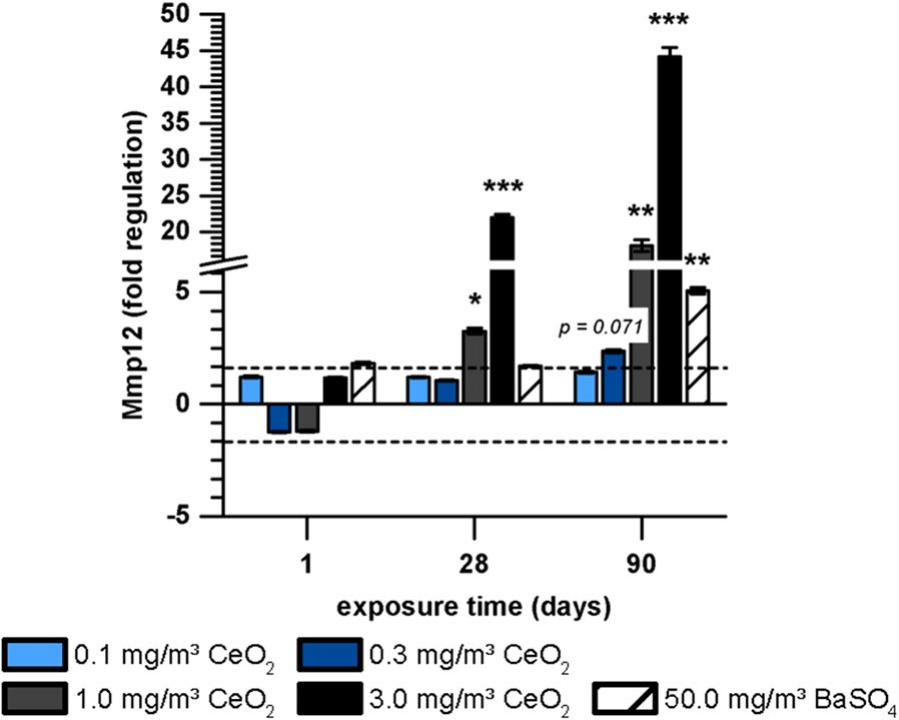
Gene expression of Mmp12 in AEII cells in response to nanoparticle exposure.Gene expression of Mmp12 in AEII cells of rats exposed to 0.1, 0.3, 1.0 or 3.0 mg/m^3^ CeO_2_ or 50.0 mg/m^3^ BaSO_4_ nanoparticles for one, 28 or 90 days is illustrated. Values are expressed as mean fold regulation of clean air control ± SD; cut-off: fold regulation ≤ − 2.0 or ≥ 2.0 (dotted line), *p < 0.05, **p < 0.01, ***p < 0.001; n ≤ 5; Student’s T-test analysis of the replicate 2^−∆Ct^ values for each gene in the control and treatment groups

The lung cancer pathway revealed seven regulated genes (Table 2). The strongest response was detected for Mmp12 (Fig. 5). Fold regulation values markedly increased with ongoing CeO_2_ nanoparticle exposure and increasing concentration. BaSO_4_ effects were lower, but still caused significant upregulation of Mmp12 after 90 days.

### Analysis of protein expression in the alveolar compartment

For the pathways inflammation, oxidative stress and lung cancer, the following regulated genes were investigated regarding their protein expression in either BAL or lung tissue of CeO_2_ and BaSO_4_ exposed animals: Ccl2, Ccl20, Il-1α and Il-1ß (BAL analysis), Lpo and Mmp12 (immunohistochemistry of lung tissue).

#### Ccl2 levels in bronchoalveolar lavage

**Fig. 6:**
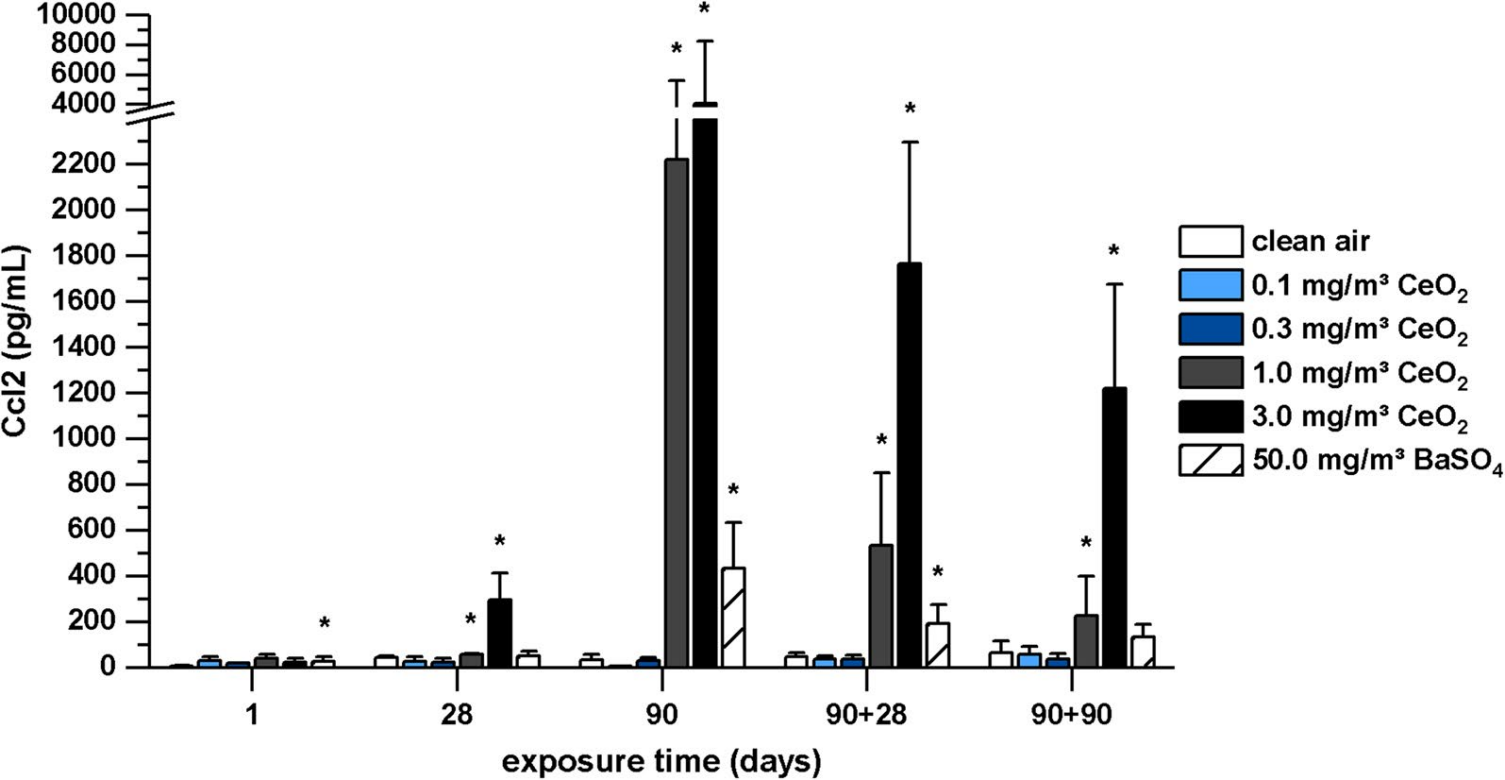
Ccl2 levels measured in bronchoalveolar lavage. Rats were exposed to clean air, 0.1, 0.3, 1.0, and 3.0 mg/m^3^ CeO_2_ nanoparticles or 50.0 mg/m^3^ BaSO_4_ nanoparticles. Ccl2 levels were determined after one, 28 and 90 days exposure and 28 and 90 days after the end of 90 days exposure (90 + 28, 90 + 90). Values are expressed as mean + SD, *p ≤ 0.05 vs. clean air control, n ≤ 5; Kruskal–Wallis–ANOVA with Mann–Whitney U-test as post hoc analysis

While Ccl20, IL-1α, and IL-1β protein levels in BAL did not change in response to nanoparticle exposure, Ccl2 was markedly increased with ongoing CeO_2_ exposure and increasing concentration (Fig. 6). In addition to gene expression analysis, mediator levels in BAL were also measured during post-exposure of up to 90 days. Although a slight decrease of Ccl2 levels was observed in this phase, elevated protein expression was overall persistent. BaSO_4_ also significantly affected Ccl2 protein expression in BAL.

#### Immunohistochemical analysis of Lpo and Mmp12

**Fig. 7:**
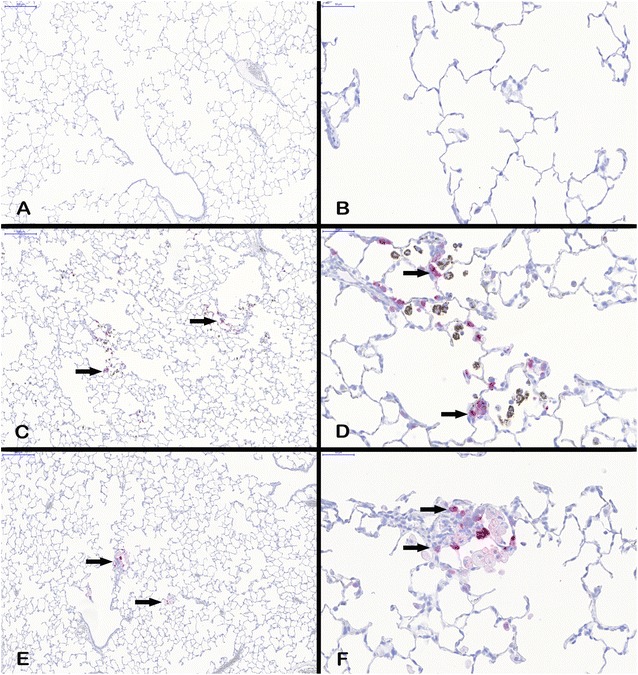
Lpo protein expression in lung tissue. All examples illustrate findings after 90-day nanoparticle exposure. **A** lung tissue overview, ×10, and **B** detailed view, ×40, after clean air exposure, **C** Lpo positive cells (arrows) in lung tissue overview, ×10, and **D** detailed view, ×40, after 3.0 mg/m^3^ CeO_2_ exposure, and **E** Lpo positive cells (arrows) in lung tissue overview, ×10, and **F** detailed view, ×40, after 50.0 mg/m^3^ BaSO_4_ exposure. The images show Lpo-positive cells counterstained with hematoxylin

**Fig. 8:**
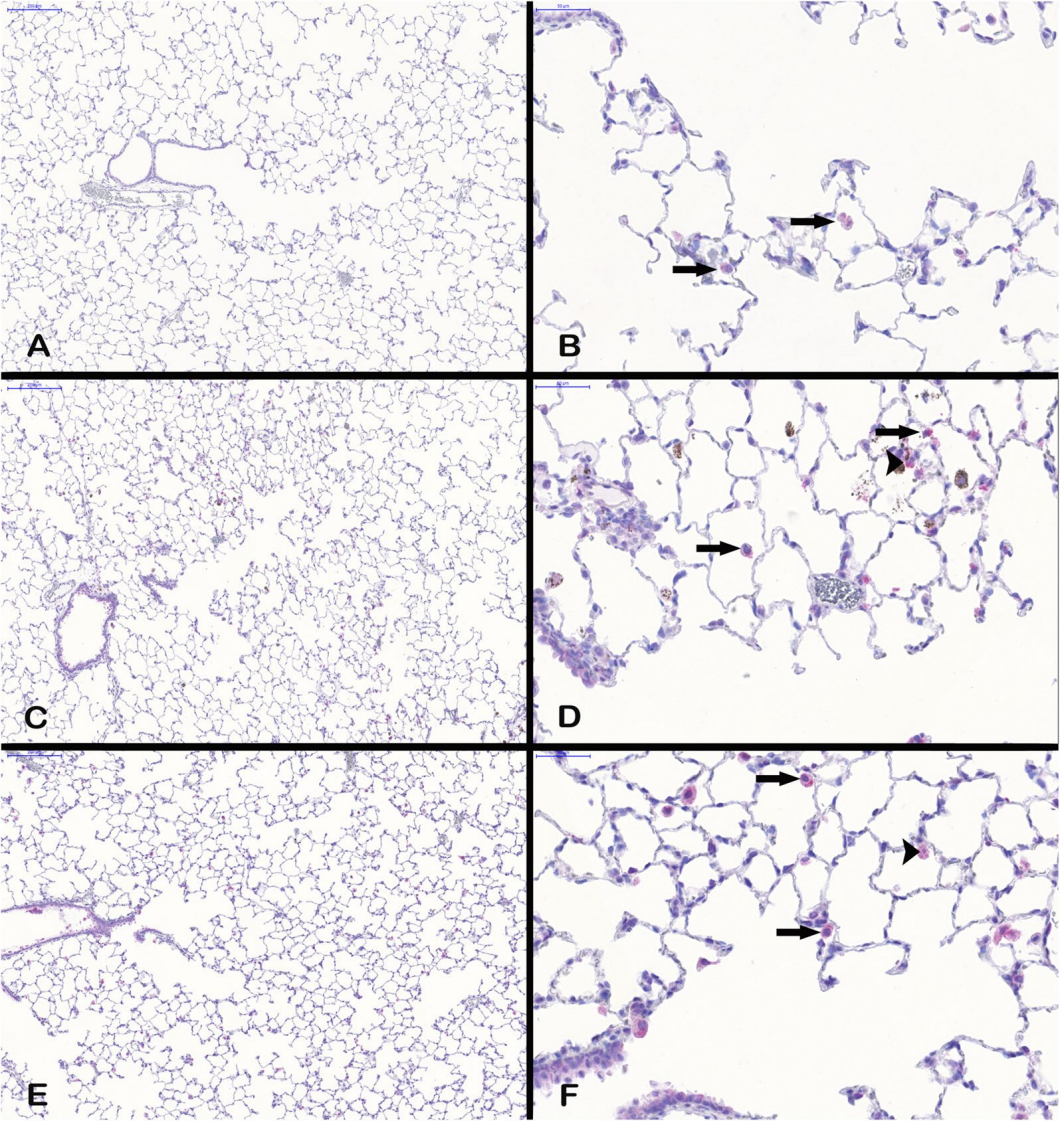
Mmp12 protein expression in lung tissue. All examples illustrate findings after 90-day nanoparticle exposure. **A** Mmp12 positive macrophages (arrows) in lung tissue overview (×10) and **B** detailed view (×40) after clean air exposure, **C** Mmp12 positive macrophages (arrows) and AEII cells (arrowhead) in lung tissue overview (×10) and **D** detailed view (×40) after 3.0 mg/m^3^ CeO_2_ exposure, and **E** Mmp12 positive macrophages (arrows) and AEII cells (arrowhead) in lung tissue overview (×10) and **F** detailed view (×40) after 50.0 mg/m^3^ BaSO_4_ exposure. The images show Mmp12-positive cells counterstained with hematoxylin

**Fig. 9:**
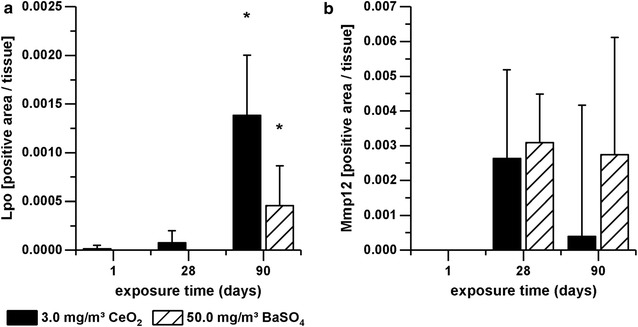
Lpo and Mmp12 levels in alveolar tissue. Rats were exposed to clean air, 3.0 mg/m^3^ CeO_2_ nanoparticles or 50.0 mg/m^3^ BaSO_4_ nanoparticles. **a** Lpo and **b** Mmp12 levels were determined immunohistochemically after one, 28 and 90 days exposure. Values are expressed as positive area per total tissue, normalized to the control group, mean + SD, *p < 0.05 vs. clean air control, n ≤ 5; Kruskal–Wallis–ANOVA with Mann–Whitney U-test as post hoc analysis

**Fig. 10:**
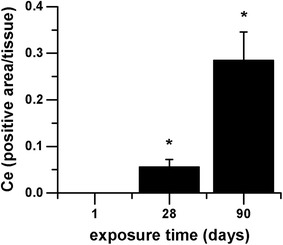
Cerium levels in alveolar tissue. Rats were exposed to clean air or 3.0 mg/m^3^ CeO_2_ nanoparticles. Cerium levels were determined in tissue slices after one, 28 and 90 days exposure. Values are expressed as positive area per total tissue, normalized to the control group, mean + SD, *p < 0.05 vs. clean air control, n ≤ 5; Kruskal–Wallis–ANOVA with Mann–Whitney U-test as post hoc analysis

Since gene expression of Lpo and Mmp12 was highly upregulated in AEII cells, immunohistochemistry was used to evaluate the development of their protein level. Figures 7 and 8 exemplarily show tissue sections of the alveolar region of rats exposed to clean air, 3.0 mg/m^3^ CeO_2_, or 50.0 mg/m^3^ BaSO_4_ for 90 days. The red colored signal indicates increased protein expression of Lpo (Fig. 7) or Mmp12 (Fig. 8) in response to both materials, while clean air did not cause any response. Lpo was detected in the alveolar epithelia in close proximity to the opening of the terminal bronchioli in regions of accumulated particle-laden macrophages and induced inflammation. The Lpo positive cells were morphologically consistent with AEII cells. Furthermore, no significant levels were measured in alveolar macrophages. This indicates that the increased Lpo gene expression in AEII cells caused also increased Lpo protein levels in the same cells. Lpo positive areas were additionally quantified per total tissue area. This analysis revealed an increasing signal with ongoing nanoparticle exposure, significant after 90 days for both substances (Fig. 9). The effect of CeO_2_ was higher compared to BaSO_4_ according to gene expression results. This is also visible in Fig. 7. The lung tissue overview sections contain more areas of CeO_2_ particle-laden macrophages with positive Lpo signal in epithelial cells (Fig. 7C) than BaSO_4_ related signals (Fig. 7E). As seen in Fig. 7D, the brown colored CeO_2_ particles accumulated in macrophages are histologically visible. The analyzed tissue was therefore further used for quantification of cerium (Fig. 10). The signal was significantly exceeding control levels and was increasing with ongoing particle exposure.

Mmp12 protein levels were increased after 28 and 90 days nanoparticle exposure, but did not yield any significance, due to high variation between individual tissue slides (Fig. 9b). The picture of the lung sections (Fig. 8) indicates that the positive signal originates predominantly from alveolar macrophages rather than epithelial cells. However, single Mmp12 positive AEII cells were also detectable.

### Gene expression analysis of liver and kidney tissue

**Fig. 11:**
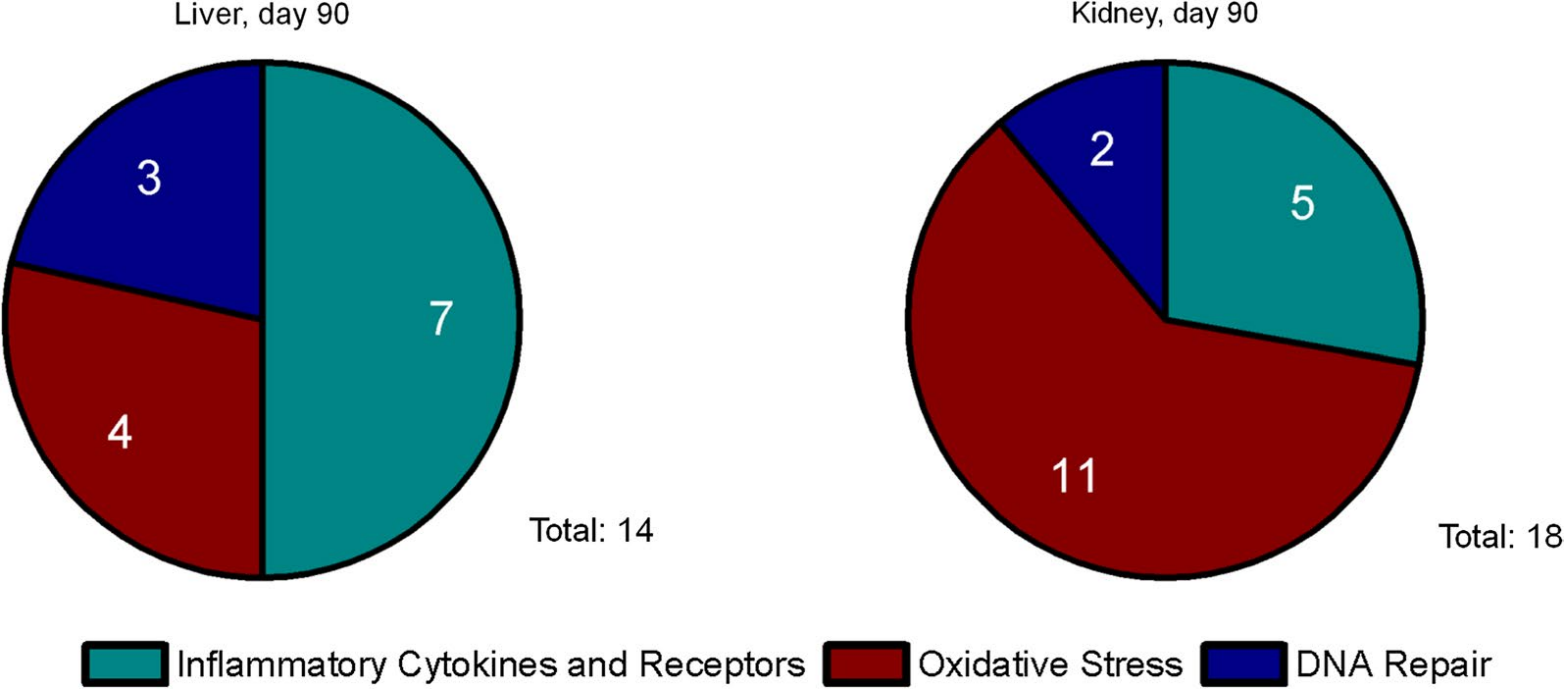
Number and distribution of regulated genes in liver and kidney. The number of regulated genes in liver and kidney tissue after 90-day nanoparticle exposure is illustrated per pathway (pool of all treatment groups)

In liver and kidney tissue 14 and 18 genes respectively were regulated in total (Table 3, Additional file 1: Table S1). Similar to the AEII cells a high response concerning the amount of regulated genes was seen for the endpoint inflammation (50% of total for liver, ~ 30% of total for kidney; Fig. 11). However, in kidney tissue even more genes were found to be related to oxidative stress, most of them downregulated. In both tissues a minor portion of the regulated genes was DNA repair-related. Comparison of both substances indicated a higher response of gene regulation to CeO_2_ than to BaSO_4_.

**Table 3:** Gene regulation in liver and kidney in response to 90 days CeO_2_ and BaSO_4_ exposure

Array	Gene		CeO_2_		BaSO_4_	
			Liver	Kidney	Liver	Kidney
Inflammatory cytokines and receptors	Ccr2	Chemokine (C–C motif) receptor 2				
	Cx3cl1	Chemokine (C–X3–C motif) ligand 1				
	Cxcl12	Chemokine (C–X–C motif) ligand 12				
	Cxcr2	Chemokine (C–X–C motif) receptor 2				
	Cxcr3	Chemokine (C–X–C motif) receptor 3				
	Il2rg	Interleukin 2 receptor, gamma				
	Osm	Oncostatin M				
	Ccl2	Chemokine (C–C motif) ligand 2				
	Ccl9	Chemokine (C–C motif) ligand 9				
	Cxcr5	Chemokine (C–X–C motif) receptor 5				
	Spp1	Secreted phosphoprotein 1				
Oxidative stress	Gpx2	Glutathione peroxidase 2				
	Lpo	Lactoperoxidase				
	Ncf2	Neutrophil cytosolic factor 2				
	Scd1	Stearoyl-Coenzyme A desaturase 1				
	Alb	Albumin				
	Epx	Eosinophil peroxidase				
	Gclc	Glutamate-cysteine ligase, catalytic SU				
	Gclm	Glutamate cysteine ligase, modifier SU				
	Hba1	Hemoglobin alpha, adult chain 2				
	Mpo	Myeloperoxidase				
	Ngb	Neuroglobin				
	Txnip	Thioredoxin interacting protein				
DNA repair	Lig4	Ligase IV, DNA, ATP-dependent				
	Smug1	Single-strand-selective monofunctional uracil-DNA glycosylase 1				
	Xrcc2	X-ray repair complementing defective repair in Chinese hamster cells 2				
	Brca1	Breast cancer 1				
Total	27		13	17	6	6

upregulation;  downregulation;  up- or downregulation (differences between dose groups); cut-off: FR ≤ − 2.0 or ≥ 2.0

### Nanoparticle retention in liver and kidney tissue

**Fig. 12:**
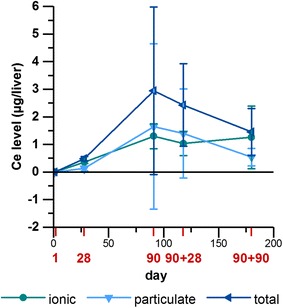
Cerium level in liver tissue after CeO_2_ nanoparticle exposure. Ionic, particulate and total Ce levels (µg) were determined in liver tissue after one, 28 and 90 days exposure and 28 and 90 days after the end of 90 days exposure to 3.0 mg/m^3^ CeO_2_ nanoparticles. Values are expressed as mean ± SD

Since some changes in gene expression were detected in liver and kidney tissue after nanoparticle exposure, we determined the amount of Ce in both organs. Figure 12 exemplary shows the level of ionic, particulate and total Ce in the liver during and after 90 day exposure to 3.0 mg/m^3^ CeO_2_. The overall amount is low, but there is a significant increase in Ce levels with ongoing exposure and values decrease after the last day of exposure. Differences in the level of ionic and particulate Ce are marginal. A switch from more ionic to more particulate Ce with ongoing nanoparticle exposure and back to higher ionic levels during post-exposure was detected. Table 4 contains all Ce levels measured in liver and kidney for the mid and high dose group. 1.0 mg/m^3^ CeO_2_ caused slightly lower values and a similar trend as seen in the high dose group, with peak levels of 2 µg/liver after 90 days exposure. The lower Ce dose groups and BaSO_4_ were not measured at this point.

**Table 4:** Amounts of Ce in liver and kidney tissue after CeO_2_ nanoparticle exposure

Dose group	Ce	Ce content (µg/organ ± SD)				
		Day 1	Days 28	Days 90 + 1	Days 90 + 28	Days 90 + 90
4 (liver)	Ionic	0.025 ± 0.001	0.27 ± 0.068**	0.717 ± 0.407**	0.68 ± 0.38**	0.46 ± 0.70**
	Particulate	0.054 ± 0.001	0.081 ± 0.04**	1.308 ± 1.094**	1.13 ± 1.58**	0.42 ± 0.24*
	Total	0.079 ± 0.002	0.34 ± 0.049**	2.025 ± 1.182**	1.81 ± 1.78**	0.88 ± 0.60*
5 (liver)	Ionic	< 0.025	0.35 ± 0.038**	1.30 ± 0.45**	1.03 ± 0.43**	1.26 ± 1.13**
	Particulate	< 0.025	0.13 ± 0.080	1.65 ± 3.00*	1.40 ± 1.61**	0.54 ± 0.31**
	Total	< 0.025	0.48 ± 0.078**	2.95 ± 3.04**	2.43 ± 1.50**	1.45 ± 0.85**
4 (kidney)	Ionic	0.11 ± 0.06	0.11 ± 0.07	0.04 ± 0.01	0.04 ± 0.01	0.05 ± 0.03
	Particulate	0.04 ± 0.03	0.07 ± 0.03	0.15 ± 0.10*	0.07 ± 0.02	0.05 ± 0.02
	Total	0.15 ± 0.07	0.18 ± 0.10	0.19 ± 0.09	0.11 ± 0.02	0.09 ± 0.05
5 (kidney)	Ionic	0.018 ± 0.001	0.025 ± 0.000	0.032 ± 0.012**	0.041 ± 0.009**	0.059 ± 0.015**
	Particulate	0.025 ± 0.010	0.021 ± 0.006	0.030 ± 0.003	0.033 ± 0.029	0.042 ± 0.02
	Total	0.029 ± 0.011	0.033 ± 0.018	0.050 ± 0.019	0.073 ± 0.033*	0.084 ± 0.038*

Dose groups: 4 = 1.0 mg/m^3^ CeO_2_, 5 = 3.0 mg/m^3^ CeO_2_; Values are expressed as mean ± SD, * p < 0.05, ** p < 0.01 vs. d 1, n ≤ 5; Kruskal–Wallis–ANOVA with Mann–Whitney U-test as post hoc analysis; detection limit for analysis of liver tissue: 0.025 µg; detection limit for analysis of kidney tissue: 0.015 µg

## Discussion

Gene expression analysis is a highly sensitive method, which can yield valuable supportive information on a mechanistic level. Therefore, the gene expression analysis described here was performed as extension of a 90-day inhalation toxicity study on nanomaterial according to OECD TG 413. It aimed on the identification of early biomarkers predicting effects of subacute to chronic nanoparticle exposure. Results of the guideline required parts of the study are discussed in detail elsewhere [1]. Briefly, a marked pulmonary inflammation was detected in response to 3.0 mg/m^3^ CeO_2_ nanoparticle inhalation, showing increasing severity with ongoing particle exposure and post-exposure persistency. This particle concentration most likely provoked lung overload, indicating that the inflammation was caused by oversaturated macrophages and related impaired lung clearance. Histopathological findings suggest the risk of long-term effects like fibrosis, resulting from the chronic inflammation reaction. For the mid concentration (1.0 mg/m^3^ CeO_2_) also signs of inflammation accompanied by slightly impaired lung clearance were detected. Depending on the calculation method, overload is assumed to be present or absent and therefore making a clear conclusion difficult. At lower CeO_2_ concentrations (0.1 and 0.3 mg/m^3^) no signs of inflammation and overload were detected. BaSO_4_ nanoparticles (50.0 mg/m^3^) also caused mild inflammatory cell infiltrations in the alveolar space, although major effects were restricted to the nasal cavity, due to particle accumulation. Interestingly, despite the high concentration administered to the animals, BaSO_4_ was cleared rapidly from the lung without provoking overload.

### Local alveolar effects of CeO_2_ nanoparticles

An overview of CeO_2_ nanoparticles’ influence on the alveolar compartment in terms of inflammation, oxidative stress and tissue degradation is depicted in Fig. 15.

The described inflammation was reflected by gene expression analysis. In AEII cells a total number of 34 genes were regulated after nanoparticle exposure and thereof 18 were chemokines or interleukins, responsible for immune cell activation and recruitment. This matches inflammatory cell infiltrations in lung tissue and increasing neutrophil and lymphocyte levels in BAL detected in the main study [1]. A major part of the effects exacerbate time- and concentration-dependent, which was reflected by two different phenomena on mRNA level: (1) increase in total number of regulated genes, (2) increase in fold regulation of the individual genes.

**Fig. 13:**
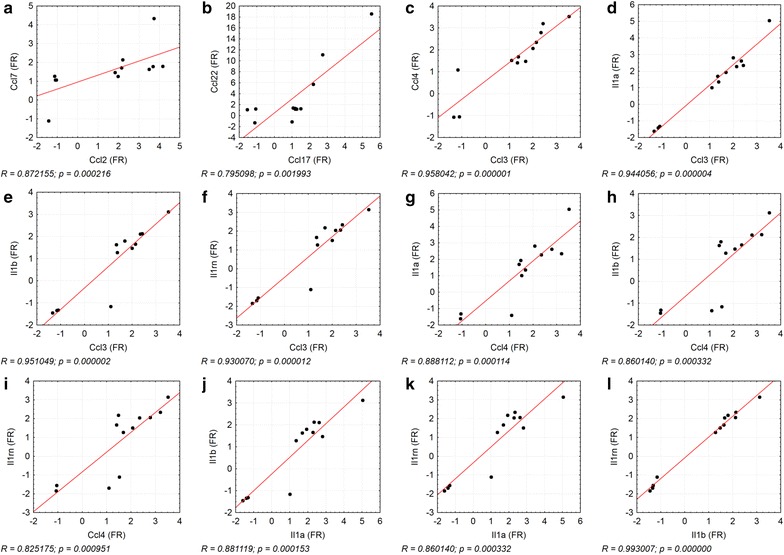
Correlation between fold regulations of chemokines. The correlation between gene expression fold regulation (FR) values of **a** Ccl2/Ccl7, **b** Ccl17/Ccl22, **c** Ccl3/Ccl4, **d** Ccl3/Il1a, **e** Ccl3/Il1b, **f** Ccl3/Il1rn, **g** Ccl4/Il1a, **h** Ccl4/Il1b, **i** Ccl4/Il1rn, **j** Il1a/Il1b, **k** Il1a/Il1rn, and **l** Il1b/Il1rn is illustrated. The individual data points display the FR values of each dose groups 0.1, 0.3, 1.0, and 3.0 mg/m^3^ CeO_2_ per time point (day 1, 28, 90). R = Spearman’s correlation coefficient, level of significance p ≤ 0.05; Spearman’s rank correlation analysis

Pulmonary immune responses imply the complex interaction between different inflammatory cell types, but also respiratory epithelial cells. In particular AEII cells are suggested to release inflammatory mediators. Most distinct regulation in these cells was detected for the C–C motif chemokines Ccl2, Ccl7, Ccl17 and Ccl22 after 1.0 and 3.0 mg/m^3^ CeO_2_ nanoparticle exposure. Ccl2 and Ccl7 are closely related chemoattractant proteins, which bind to the same receptor (Ccr2) and cause similar responses, like macrophage, lymphocyte and neutrophil recruitment [44]. Ccl17 and Ccl22 also react via a common receptor (Ccr4). They are involved in activation of CD4+ T-lymphocytes and monocytes [45, 46]. Our results show upregulation of all these chemokines in response to CeO_2_ nanoparticle exposure and correlations between the related mediators Ccl2/Ccl7 and Ccl17/Ccl22 (Fig. 13a, b). At exposure conditions resulting in high gene regulation (90 days exposure, CeO_2_ concentration ≥ 1.0 mg/m^3^) also significant changes in the amount of target cells for those mediators have been detected in histopathology and/or BAL (particle-laden macrophage accumulations, increased lymphocyte levels) [1]. Until now less was known regarding the expression of the mentioned chemokines in AEII cells in general or in response to nanoparticles. Several studies indicate Ccl2 production in isolated AEII cells after different stimuli [3, 47–52]. Cxcl2 or Ccl2 mRNA expression in either isolated AEII cells or alveolar epithelial cell lines has been described already in response to quartz exposure [6, 53, 54] and Chen et al. [8] demonstrated the expression and release of different Cxcl-motif chemokines by AEII cells after intra-tracheal instillation of carbon nanoparticles and assumed AEII cells to be the major key player in this neutrophil-driven response.

Lower, non-overload concentration levels should help to evaluate potential substance related effects. Our previous investigations indicated that at a concentration of 1.0 and even 3.0 mg/m^3^ CeO_2_ effects are likely not exclusively overload-related but also dependent on material-specific characteristics, because they did not induce a volumetric or specific surface-related lung overload [1]. The upregulation of chemokines is therefore to some extent substance related either. CeO_2_ nanoparticle exposure at 0.1 and 0.3 mg/m^3^ revealed gene regulation of inflammatory mediators, not markedly affected at higher dose levels (Ccl3, Ccl4, Il-1α, Il-1β, Il-1rn). Ccl3 and Ccl4 are two related mediators with the shared receptor Ccr5. They function as chemoattractant for mainly monocytes and lymphocytes and are expressed by a variety of cells, including inflammatory and epithelial cells [55]. Il-1α and Il-1ß both bind to the interleukin receptor and trigger chemokine release from target cells and immune cell activation. The antagonist Il-1rn competes with Il-1α and Il-1ß for interleukin receptor binding and by this balances induced immune reactions. Evidence is given that Il-1α and Il-1ß stimulate Ccl2 expression in AEII cells [3, 4, 51, 56]. Brabcová et al. [57] demonstrated in addition to Ccl2 effects on Ccl3, Ccl4, and Ccl17 levels in A549 cell cultures in response to Il-1ß and graded this interleukin as a quite potent chemokine stimulant. Il-1ß synthesis has also been found in stem cell-derived lung epithelial cells [58] and isolated mouse AEII cells exposed to TiO_2_ nanoparticles [59]. So far, data on the influence of nano-CeO_2_ on the release of the discussed mediators by AEII cells has not been reported by others. Only some measurements of Il-1ß have been performed in BAL [19, 26] or lung tissue [30] of rodents treated with CeO_2_. However, since no cell type specific analysis was performed the cellular source of Il-1ß remains unknown.

90 days exposure to concentrations of up to 0.3 mg/m^3^ revealed significant upregulation of the respective mediators followed by a decrease to baseline levels in higher dose groups and highly correlated expression patterns between all mediators (Fig. 13c–l). Since the chemokines and interleukins indicate inflammation which was for this concentration not yet measureable in BAL analysis but clearly present at higher dose levels (BAL and histopathology) [1], the respective mediators might function as promising sensitive biomarkers for nanoparticle exposure on a gene expression level. Also, this gene regulation might indicate an early risk of induced pulmonary inflammation during long-term low dose exposure to CeO_2_ nanoparticles. In parallel to our 90-day study respective investigations will be provided by a long-term setup with the same CeO_2_ nanomaterial and concentrations as part of the European program on the regulatory testing of manufactured nanomaterials (NANoREG), in which potential toxic and carcinogenic effects during and after exposure periods of up to 1 year were investigated [BASF, Ludwigshafen, Germany, “combined chronic inhalation toxicity and carcinogenicity study with CeO_2_ and BaSO_4_”, (81|0661/10|170)]. The combination of both studies constitutes the ideal base to prove and classify the meanings of the potential early signs for inflammatory effects discussed here.

It could be assumed that AEII cells support the defense against nanoparticles in second instance since upregulation of inflammatory mediators started at later stages of exposure and was dominated by chemokines stimulating monocytes and cells of the adaptive immune system (e.g. lymphocytes). Constant monocyte recruitment supports elimination of the rising particle amount and handling of overload conditions, lymphocytes are usually recruited for delayed defenses. Our histopathology and BAL analysis match this theory as we found neutrophilic inflammation at early stages of exposure, followed by increasing levels of lymphocytes [1]. Respective observations were accompanied by sustained presence of particle-laden macrophages.

Although inflammatory effects of CeO_2_ nanoparticle exposure were dominating signs of induced oxidative stress have additionally been detected. A marked time-dependent upregulation of Lpo mRNA and protein was measured in AEII cells. Protein analysis further identified the increased Lpo levels as being related to areas of particle-laden macrophage accumulations and inflammatory cell infiltrations. Lpo catalyzes the conversion of thiocyanate to antibacterial hypothiocyanite by use of hydrogen peroxide and belongs to the non-immunological airway defense system. The respiratory Lpo defense system highly depends on H_2_O_2_ and is influenced by the presence of inflammatory mediators [60]. Based on research with oxidized SWCNT evidence is given that Lpo functions in nanomaterial degradation [61]. Since CeO_2_ particles bear redox activity, an interaction with the Lpo defense system seems reasonable and supports substance related effects. The particles might be able to process H_2_O_2_ by Ce^3+^/Ce^4+^ redox cycling. Although respective properties were reviewed from an anti-oxidative perspective, in terms of mimicking superoxide dismutase (SOD) or catalase activities, CeO_2_ obviously has the potential to affect H_2_O_2_ balance. The SOD like reaction includes the generation of H_2_O_2_ from superoxide anion. This would favor the increase of extracellular H_2_O_2_ and might cause elevated Lpo levels. Also other theories of oxidative stress generation, including lipid peroxidation [19] by the particles are conceivable. The catalytic activity thus claims consideration of both, pro- and anti-oxidative capacities.

**Fig. 14:**
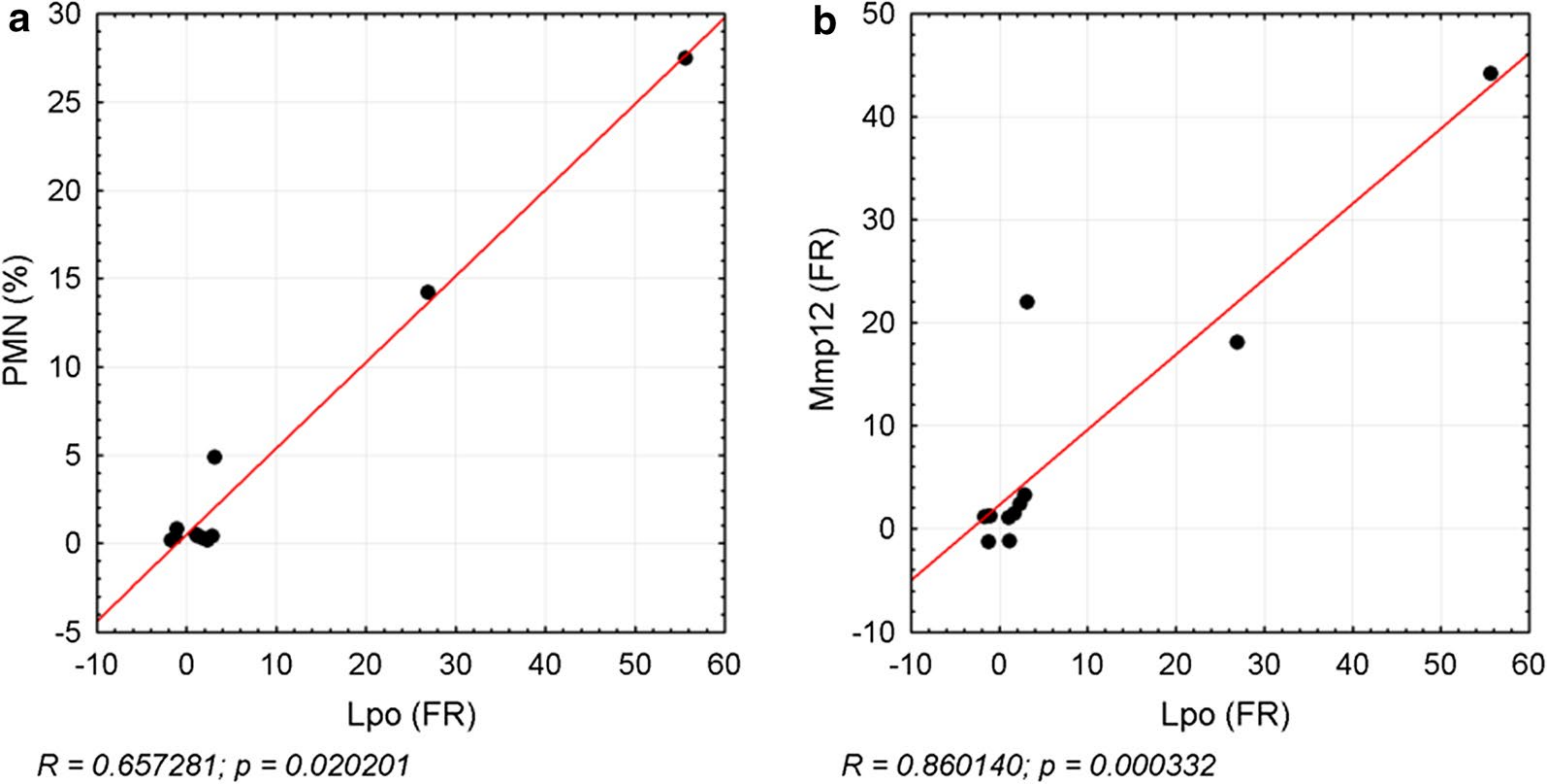
Correlation of Lpo to neutrophils and Mmp12 expression. The correlation of Lpo gene expression fold regulation (FR) values to the percentage of PMN levels in **a** BAL, and the FR of **b** Mmp12 is illustrated. Data points of the dose groups 0.1, 0.3, 1.0, and 3.0 mg/m^3^ CeO_2_ for each time point (day 1, 28, 90) are depicted. R = Spearman’s correlation coefficient, level of significance p ≤ 0.05; Spearman’s rank correlation analysis

Furthermore, an inflammation-related hypothesis for elevated Lpo levels could be based on respiratory burst. Inflammatory cells eliminate internalized material by the production of ROS. Neutrophils for instance release O_2_^−^ and H_2_O_2_ in response to various stimuli [62]. Lung epithelial cells are further known to physiologically produce H_2_O_2_ [63]. Bronchial epithelial cells have been shown to increase H_2_O_2_ release in response to the inflammatory mediator IFNγ, leading to stimulation of the Lpo defense system [60]. Such conditions might thus be responsible for imbalance of the system during inflammation (1.0 or 3.0 mg/m^3^ CeO_2_), manifested by increased Lpo levels in AEII cells at hotspots of inflammation and the correlation between the amount of neutrophils in BAL and Lpo gene expression levels (Fig. 14a).

Another upregulated factor participating in ROS generation is Noxo1, which is part of the transmembrane Nox enzyme complex and contributes to extracellular ROS production by intracellular consumption of NADPH [64]. It was significantly upregulated in response to 3.0 mg/m^3^ CeO_2_ when also a marked Lpo expression was observed. Van Klaveren et al. [65] demonstrated activation of a membrane bound NAPDH oxidase-like system in isolated rat AEII cells and enzymatic generation of O_2_^−^ and H_2_O_2_ in response to different stimuli. The potential contribution of this system to H_2_O_2_ levels suggests a functional relationship between Noxo1 and Lpo levels after CeO_2_ nanoparticle exposure.

No clear evidence was given for effects on genotoxicity and apoptosis in AEII cells after CeO_2_ nanoparticle exposure since no distinct gene regulation was measured for the tested DNA repair- or apoptosis-related genes.

However, we further found some indications for chronic particle related effects, including fibrosis and lung cancer. Mmp12 plays an important role in lung tissue remodeling and serves as marker for related acute and chronic lung diseases like fibrosis and COPD [66, 67]. Activated alveolar macrophages produce Mmp12 for neutrophil activation and release of neutrophil elastase. This leads to tissue degradation and finally emphysema development. The persistent neutrophil infiltrations measured during our previous investigations [1], accompanied by increased Mmp12 mRNA in lung epithelia indicate such mechanisms and the risk of developing chronic pathological changes during long-term exposure. Although immunohistochemical staining of lung tissue for Mmp12 did not reveal significantly elevated protein levels, Mmp12 might serve as a valid marker for substance related mechanisms of action as mRNA upregulation occurs at absent lung overload with CeO_2_ nanoparticles. Overexpression of Mmp12 has been described in mouse lungs after application of asbestos or TiO_2_ nanoparticles [68, 69]. The latter caused parallel upregulation of Ccl2, 3, 4 and 7, which were also affected in our studies. Ma et al. [16] demonstrated the influence of CeO_2_ nanoparticles on the development of fibrosis by induction of epithelial–mesenchymal transition of AEII cells after intra-tracheal instillation. Interestingly, disturbance of the Lpo defense system was demonstrated in relation to the pathology of cystic fibrosis [70]. The high correlation of both mediators (Fig. 14b) therefore might function as marker for fibrosis induction in AEII cells triggered through the early inflammation events. This hypothesis is further supported by very slight interstitial fibrosis measured in the main study (90 days post-exposure, 3.0 mg/m^3^ CeO_2_) [1].

**Fig. 15:**
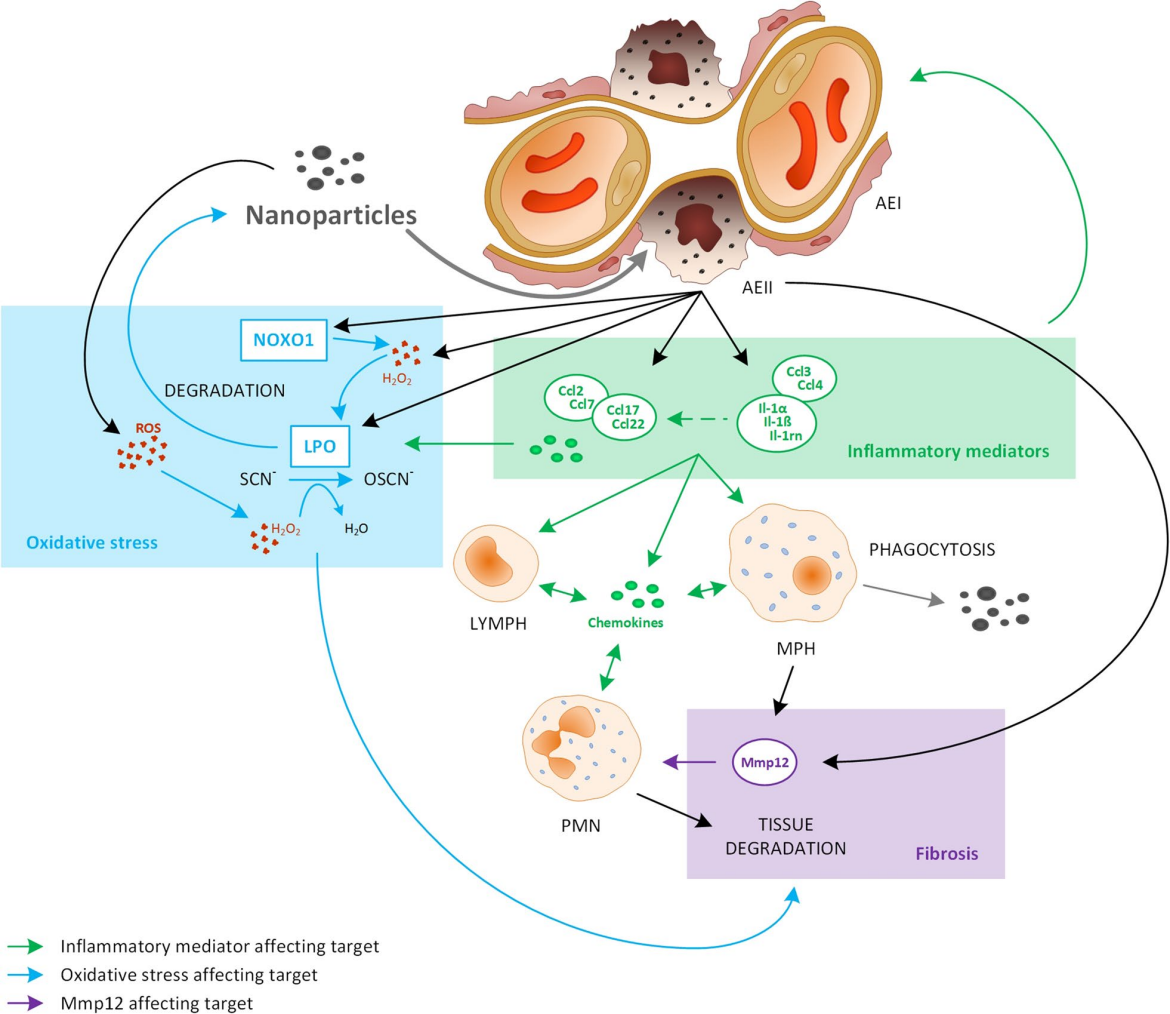
Potential mechanistic relationship between the mediators regulated in AEII cells after CeO_2_ nanoparticle exposure

Figure 15 summarizes the potential relationship between the analyzed mediators and different cell types in response to nanoparticle exposure. Increased gene expression in AEII cells is assumed to affect inflammatory cells and the lung’s oxidative balance. This might further lead to tissue degradation and long-term effects like fibrosis. Lpo, Mmp12 and the inflammatory mediators Ccl2, 7, 17, 22, 3, 4, Il-1α, Il-1β and Il-1rn are promising marker genes for the mentioned effects.

### Local alveolar effects of BaSO_4_ nanoparticles

Considering BaSO_4_ classification as chemically inert and non-toxic, a generally low and non-adverse response was expected. Therefore BaSO_4_ NM-220 was selected as reference material without any specific toxicity in the NANoREG long-term study performed by BASF (81|0661/10|170). We measured effects less severe compared to CeO_2_, despite the at least 16-fold higher concentration, which supports the low effect hypothesis. However, some unexpected findings in BaSO_4_ exposed lungs have been detected and need to be evaluated.

Pulmonary inflammation and some changes in AEII cell gene expression levels were measured. Major histopathological findings in the nasal cavity with minor inflammation of the alveolar compartment were attributed to particle deposition favoring the upper respiratory tract rather than the alveoli because of high particle accumulation [1]. This might be the reason for the lower impact on gene regulation in AEII cells compared to CeO_2_.

Interestingly, the majority of genes affected by BaSO_4_ were also affected by CeO_2_. Similar to CeO_2_ the highest response in the group of inflammatory mediators was measured for the C–C-motif chemokine Ccl22. In vitro data on the influence of BaSO_4_ on macrophages or neutrophils regarding expression of inflammatory mediators and cell migration did not show activation of respective mechanisms [71, 72]. Particles like silica and TiO_2_ in contrast, had stimulating effects [72]. All in all, the impact of BaSO_4_ on inflammatory mediator release compared to CeO_2_ is rather low. Post-exposure decrease of Ccl2 protein levels in BAL supported by decreases in severity of histopathological findings after the end of 90 days exposure [1] further suggest reversibility of the inflammatory reaction.

Although BaSO_4_ NM-220 was demonstrated to bear no intrinsic oxidative potential [73], the oxidative stress marker Lpo was highly upregulated. Like for CeO_2_ a marked increase of Lpo protein in AEII cells was identified in areas of inflammation. This verifies a mechanistic relationship between the particle-induced inflammation and oxidative stress. Further, the quantification of the Lpo signal displayed a lower, but significant elevation in comparison to CeO_2_.

Signs for genotoxicity and apoptosis were minimal, but a strong effect of BaSO_4_ nanoparticle exposure on Mmp12 was detected. Considering the relation of Mmp12 to chronic pulmonary effects like fibrosis, potential adverse reactions due to long-term BaSO_4_ become more likely. However, such findings were not detected in previous histopathological investigations [1]. Also, Mmp12 protein levels in lung tissue remain unaffected. Since Mmp12 further indicates inflammation, the elevated gene expression levels in AEII cells detected for BaSO_4_ might be related to the inflammatory reaction without notifying chronic adverse effects.

Although BaSO_4_ was cleared from the respiratory tract quite rapid and no overload situation was provoked during 90-day particle exposure [1], the very high amount of particles entering the respiratory tract might be a reason for detection of unexpected effects like increased Lpo, Ccl22 or Mmp12. The phenomenon of fast clearance was pointed out in our previous research [1]. The dissolution of BaSO_4_ nanoparticles is assumed to be generally low. However, according to Konduru et al. [74], who stated a dissolution rate of < 1% in different media, the fast clearance could be attributed to changes in the dissolution rate because of e.g. switches in surface charge of the nanoparticles. Nevertheless an explicit reason for the phenomenon of fast BaSO_4_ clearance remains unknown and needs to be further investigated. Our results further show that CeO_2_ and BaSO_4_ caused similar responses in AEII cells while the response pattern still allowed differentiation between both substances. The higher inflammatory potential of CeO_2_ was appropriately reflected. Mediators displaying quite high responses (Lpo, Ccl22, Mmp12) might be suitable markers for nanomaterial toxicity testing. To further clarify the specific function of Lpo or Mmp12 as response to CeO_2_ and BaSO_4_ nanoparticles, investigations of the respective markers after exposure to other nanoparticles with a known oxidative, genotoxic or tumorigenic potential are needed.

### Systemic effects of CeO_2_ and BaSO_4_ nanoparticles on liver and kidney tissue

Nanomaterial generally bears a certain potential to translocate to extra-pulmonary organs after inhalation and organs responsible for excretion are common targets. We investigated gene expression in liver and kidney tissue after 90 days exposure. The number of regulated genes was lower compared to AEII cells with less overlap between pulmonary and extra-pulmonary compartments. Distribution of genes over the different pathways however was similar especially in liver tissue where effects on inflammatory mediators were dominating. Interestingly in the kidney a great portion of genes associated with oxidative stress were downregulated. CeO_2_ had a higher impact than BaSO_4_ on both organs.

It has been demonstrated by Aalapati et al. [19] that a small fraction of CeO_2_ nanoparticles accumulate in liver and kidney and cause histopathological changes after inhalation in mice. The kidney was assumed to be the major extra-pulmonary target organ for nanoparticles. Nemmar et al. [30] comprehensively investigated inflammation, oxidative stress and DNA damage in extrapulmonary tissues after CeO_2_ intratracheal instillation and reported adverse findings for all endpoints in liver and kidney.

Our findings show upregulation of different C–X–C motif chemokines and receptors responsible for neutrophil recruitment in liver tissue in response to CeO_2_, indicating inflammatory reactions. In kidney tissue almost all genes affected by CeO_2_ displayed downregulation, therefore suggesting a rather anti-inflammatory and anti-oxidative effect in this organ. Since potential effects of CeO_2_ are controversial and positive health effects have been described [75], a positive impact of translocating particles to the kidneys could be conceivable.

As basis for discussion of the present findings, we exemplary determined the level of Ce in liver and kidney tissue during and after nanoparticle exposure to the higher concentrations. We found a small amount of ionic and particulate Ce in the liver, showing an expected development of increase during and decrease after exposure. In the previous study we measured peak levels of 1300 µg Ce retained per lung [1]. This amount derived from a concentration of 3.0 mg/m^3^ CeO_2_ NM-212 administered over 90 days (6 h/day, 5 days/week) at a deposition fraction of 9.6%. In the liver we found 3 µg/organ at these conditions. The fraction translocated to the liver from the amount present in the lung was thus 0.23%. However, the increase of Ce levels is significant over time and a contribution to the changes in gene expression is conceivable. It also needs to be considered that modulated gene expression can be caused by other systemically distributed mediators as a response to the present lung inflammation. Also the Ce level was slightly lower after 1.0 mg/m^3^ CeO_2_ exposure compared to 3.0 mg/m^3^ CeO_2_. This indicates a concentration-dependency which has clearly been detected in the lung as well [1]. Even lower levels are thus assumed for the lower dose groups. At earlier stages of exposure the amount of ionic Ce was higher than the particulate proportion. This indicates that translocation of particles occurs with delay due to barriers and defense mechanisms. As described earlier CeO_2_ nanoparticles likely influence the cellular balance of ROS due to redox activity. This ability is contributed to ion formation on the nanoparticle surface [75]. The amount of ionic Ce could be a result of this ion formation. The high Lpo levels detected in this study indicate oxidative stress in the lung. The amount of ionic Ce in this tissue however, was negligible [1]. The ion content could therefore not be correlated to the level of oxidative stress. In liver and kidney tissue the overall modulation of gene regulation was too low to adequately assess a potential correlation between ion formation and oxidative stress in these organs. The effects on gene expression and the amount of Ce in extra-pulmonary organs was overall very low. The question in how far these findings contribute to potential adverse effects thus remains unanswered. No significant increases of Ce levels were detected in kidney tissue. It could be assumed that the downregulation of several genes was rather caused by other signals, like endogenous mediators, than the nanoparticles. Due to the low response to BaSO_4_ in terms of gene expression after 90 days and the less clear results for CeO_2_ we did not include barium retention in our investigations at this point.

## Conclusion

Our results suggest that CeO_2_ related lung inflammation was supported by chemokine release of AEII cells and characterized by increased monocyte activation with subsequent neutrophil and lymphocyte infiltrations. Stimulation of rat AEII cells is likely not exclusively overload but also substance related. At low concentrations it is dominated by interleukins like Il-1α, Il-1ß and chemokine expression. With increasing concentrations and induction of lung overload gene regulation exacerbates and the chemokine pattern switches. AEII cells further respond with signs of oxidative stress indicated by increased Lpo and Noxo1 in the presences of CeO_2_ and the mediators can be considered as potential early marker for nanoparticle oxidative stress induction after inhalation. Genes related to genotoxicity and apoptosis were not markedly affected by CeO_2_ exposure. However, due to the upregulation of Mmp12 a relation to potential long-term effects like fibrosis and lung cancer could be assumed. Respective findings need to be related to the long-term inhalation toxicity study (BASF, Ludwigshafen, Germany) to further support significance.

BaSO_4_ nanoparticle exposure has a rather low influence on the expression of inflammatory mediators in AEII cells. However, we detected some changes in gene regulation, which supports the assumption that the nanomaterial affects the organism after inhalation although it is considered as chemically inert. Interestingly, mediators like Lpo, Ccl22 and Mmp12, highly affected by CeO_2_ exposure also responded to BaSO_4_. Such mediators are promising biomarkers for nanomaterial toxicity testing.

The overall effect on gene expression in liver and kidney was low. However, especially in the liver some inflammatory mediators were upregulated. Also, low but significant Ce levels were present in the liver. A contribution of the particles to changes in gene expression of extra-pulmonary organs therefore still needs to be taken into consideration and further research should focus on this issue.

## Methods

### Test substances

The test materials cerium dioxide NM-212 and barium sulfate NM-220 belong to the Nanomaterial Repository of the European Commission Joint Research Center (JRC, Ispra, Italy) and were provided by the Fraunhofer Institute for Molecular Biology and Applied Ecology (Schmallenberg, Germany).

### Animals

For this study female Wistar rats [Crl:WI (Han)] at the age of 10 weeks during start of exposure were used (Charles River, Sulzfeld, Germany). Beforehand, animals were acclimatized to laboratory conditions for 1 week, trained for restraint in nose-only tubes over 3 weeks and were randomly distributed to control and treatment groups, respectively. The following conditions were set for animal housing: 20–24 °C, 40–70% relative humidity, 12 h light/dark cycle. Laboratory diet (“V1534”, sniff Spezialdiaeten GmbH, Soest, Germany) and water was supplied ad libitum. All experiments and animal handling was conducted in compliance with the Regulations of the German Animal Protection Law (Tierschutzgesetz of May 18, 2006).

### Nose-only inhalation

The examinations described here were performed as additional part to the conventional investigation of a 90-day inhalation toxicity study according to OECD TG 413. For gene expression analysis, 90 animals were exposed to clean air or the test substances via nose-only inhalation for one, 28 and 90 days in concentrations of 0.1, 0.3, 1.0 and 3.0 mg/m^3^ for CeO_2_ nanoparticles and 50.0 mg/m^3^ for BaSO_4_ nanoparticles (5 rats/dose group/time point). Aerosol generation was done via dry powder dispersion using a dispersion nozzle developed at Fraunhofer ITEM and the respective aerosol generation system [76]. Aerosol concentrations were constantly checked via an aerosol photometer (Fraunhofer ITEM, Hannover, Germany). The MMAD of the samples was determined by gravimetry (Marple 298 Personal Cascade Impactor, Thermo Fisher Scientific, Dreieich, Germany). Animals were kept in the inhalation system 5 days a week for 6 h each day. They were checked for clinical signs daily before and after exposure. Bodyweight as well as food and water consumption was recorded weekly.

### Gene expression analysis

#### Alveolar epithelial type II cell isolation and organ extraction

After one, 28 and 90 days of exposure to either clean air, the different CeO_2_ NM-212 concentrations or BaSO_4_ NM-220, rat lungs were prepared on the following day for isolation of AEII cells and subsequent gene expression analysis. The method of AEII isolation is based on the work of Richards et al. [77] and Dobbs et al. [78] and well established in our institute [79]. We further performed comprehensive methodical research on this topic in mice [80]. Minor modifications on the current protocol have been taken for the work with rats. Briefly, animals were anesthetized (Ketamine/Xylazine, 10:1; bela-pharm, Vechta, Germany/Bayer, Leverkusen, Germany) via intraperitoneal injection. For lung perfusion, the trachea was uncovered and cut to introduce a cannula for artificial ventilation. Abdominal cavity and thorax were opened and a cannula was inserted via the vena cava inferior into the right ventricle. Pulmonary perfusion was done with PBS (5 mL/min, 25 mL Biochrom, Berlin, Germany) and a peristaltic pump while the lung was constantly manually ventilated. An incision in the left atrium served as perfusate outflow. Finally, the lung was explanted for subsequent tissue digestion and cell isolation. During sacrifice the caudate lobe of the liver as well as the right kidney were removed and stored at − 80 °C for RNA isolation. The isolated lung was flushed with PBS, treated with dispase (Sigma-Aldrich, Taufkirchen, Germany) and manually dissected. Respective steps were followed by DNase digestion (Sigma-Aldrich, Taufkirchen, Germany) and passage through two nylon sieves with different mesh size (250, 60 µM). After several steps of centrifugation IgG panning (Sigma-Aldrich, Taufkirchen, Germany) was performed to remove unwanted cell types, including macrophages. One hour incubation was followed by another DNase digestion. Finally, the isolated cells were separated by centrifugation and the pellet was stored at − 80 °C for RNA isolation. AEII cell extraction from rat lungs yielded 5.3 ± 2.5 × 10^6^ cells per individual which was sufficient for subsequent processing.

#### RNA Isolation

RNA isolation from AEII cells, liver and kidney tissue was performed using the RNeasy Mini Kit (QIAGEN, Hilden, Germany) according to the manufacturer’s instructions including a DNase treatment procedure (RNase-Free DNase Set, QIAGEN, Hilden, Germany). RNA extraction and subsequent gene expression analysis was done for AEII cells after one, 28 and 90 days of nanoparticle exposure (90 samples in total). For liver and kidney tissues 90-day exposure samples were processed (in total 30 samples each). Amount and quality of RNA were measured with the NanoDrop ND-1000 Spectrophotometer, Version 3.7 (Thermo Fisher Scientific, Dreieich, Germany) and the Agilent 2100 Bioanalyzer (Agilent Technologies, Ratingen, Germany), respectively. The amount of RNA extracted from liver tissue was highest (78.4 ± 21.3 µg), followed by kidney (45.2 ± 6.5 µg) and AEII cells (15.6 ± 5.6 µg). Measurements of RNA quality yielded mean RNA integrity numbers (RIN) > 7.0 for liver, kidney and AEII cells, respectively, indicating RNA samples of good quality.

#### RT^2^ profiler PCR arrays

To screen a broad spectrum of potential effects of nanoparticle exposure, the following RT^2^ profiler PCR arrays (SABiosciences/QIAGEN, Hilden, Germany) were applied for AEII samples: inflammatory cytokines and receptors (PARN-011Z), oxidative stress (PARN-065Z), DNA repair (PARN-042Z), apoptosis (PARN-012Z), lung cancer (PARN-134Z). For RNA isolated from liver and kidney the same arrays were used except of the lung cancer array. The commercially available arrays contain qPCR assays for the analysis of 84 different genes related to the respective end points in a 96 well format (84 genes, 5 potential reference genes, 7 quality controls). The quality controls consist of a genomic DNA contamination control (1×), a reverse transcription efficiency control (3×) and a PCR efficiency control (3×). For the present study we created customized arrays, by pooling four commercial arrays in a 384 well format. The fifth array was spotted four times on a 384 well plate for simultaneous analysis of four samples. Some genes are overlapping between the pathways and therefore in total 391 different genes were analyzed per AEII RNA sample and 325 genes per RNA sample extracted from liver and kidney tissues (Additional file 2: Table S2). In a first step, cDNA synthesis was performed using the RT^2^ First Strand Kit (SABiosciences/QIAGEN, Hilden, Germany), following the manufacturer’s instructions. Subsequently, RT^2^ Profiler PCR arrays were conducted according to the manufacturer’s instructions using the real-time PCR system ViiA™7 (Applied Biosystems/Thermo Fisher Scientific Inc., Dreieich, Germany). An amount of 1.19 ng was applied per single RTqPCR assay.

#### Data evaluation

Data analysis of exported “Cycle Threshold” (C_T_) values was performed based on the comparative ∆∆C_T_ method described by Schmittgen and Livak [81], using the provided SABiosciences PCR Array Data Analysis Template Excel^®^ and web-based software (SABiosciences/QIAGEN, Hilden, Germany; https://www.qiagen.com/de/products/genes%20and%20pathways/data-analysis-center-overview-page/?UID=70f8b4af-789d-4788-af6f-b12ebd800888#). One to three stable expressed reference genes were chosen for each time point and tissue for normalization using NormFinder [82] and geNorm algorithm [83] as part of the GenEx Professional 6 Software (bioMCC, Freising, Germany). The fold regulation (FR) values generated for each gene describe the factor of up- or downregulation in a certain treatment group related versus the control group. Genes displaying FR values ≤ − 2 or ≥ 2 were considered as relevant. Genes with high C_T_ values (≥ 35) or abnormal melting curves were excluded. Statistical significance of FR values was checked with Student’s *T* test (p ≤ 0.05). C_T_ values, if normal distributed, were further checked for outliers within the group of five animals for each experimental condition and gene, according to Grubbs [84]. Respective values were eliminated if necessary, FR values were recalculated and statistical significance testing was repeated as described above. Genes with FR values ≤ − 2 or ≥ 2 were selected for further analysis concerning function and relevance in the context of the study focus, independent of their statistical significance. Already changes in individual animals are important, because a completely identical genetic background of the animals should not reasonably be assumed.

Correlation analysis was performed using fold regulation (FR) values (gene expression data) or mean percentages (BAL data, main study) of all CeO_2_ dose groups and time points for the two compared variables. Data were correlated using Spearman rank correlation analysis (p ≤ 0.05).

### Cytokine levels in bronchoalveolar lavage

Levels of the cytokines Ccl2, Ccl20, Il-1α, and Il-1β were measured in bronchoalveolar lavages (BAL) of rat lungs after one and 28 days as well as one, 28 and 90 days post-90-day exposure to clean air, the different CeO_2_ NM-212 concentrations or BaSO_4_ NM-220. BAL was performed according to Henderson et al. [85] with minor modifications. Lungs were lavaged twice using 4 mL 0.9% NaCl. For cytokine analysis 1 mL of the BAL fluid was removed. Cytokine levels were determined using a MULTI-SPOT^®^ 4 Spot Cytokine Custom Rat 4-Plex kit for the four analytes (Meso Scale Discovery, Rockville, USA), according to the manufacturer’s instructions.

### Immunohistochemistry

For immunohistological analysis, two consecutive lung tissue sections were prepared. Examinations were done in rats exposed to either clean air, 3.0 mg/m^3^ CeO_2_ NM-212 or 50.0 mg/m^3^ BaSO_4_ NM-220 for one, 28 or 90 days. Lungs were fixed in 10% formalin and trimmed based on the work of Kittel et al. [86]. To detect the presence of lactoperoxidase (Lpo, rabbit polyclonal, Acris, NBP1-87010) and matrixmetalloproteinase 12 (Mmp12, rabbit polyclonal, Abcam, ab66157) antibodies directed against those markers were applied as described by Rittinghausen et al. [87]. Immunohistochemically stained lung sections were counterstained with hematoxylin and digitalized using slide scanner (MiraxScanner, Zeiss, Germany) and quantified using image analyzing software (ZeissZen, Zeiss, Germany). Quantified Lpo and Mmp12 levels were statistically evaluated using Kruskal–Wallis–ANOVA with Mann–Whitney U-test as post hoc analysis.

### Retention analytics

Cerium contents were measured in liver and kidney tissue of five animals of the mid and high dose group after one and 28 exposure days and one, 28 and 90 days post-90-day exposure. The isotopes ^140^Ce/^142^Ce were quantified via inductively coupled plasma mass spectrometry (ICP-MS) using a quadrupole ICP-MS system (X-Serie II, Thermo Fisher Scientific). The shredded tissue was lyophilized for at least 6 h (0.37 mbar). Prior and subsequently to freeze-drying organ weights were recorded. Plasma ashing (cool plasma conditions, 400 W, 1 mbar O_2_, 24 h) and subsequent microwave digestion (H_2_SO_4_, 96%, supra quality, max. 500 W) was performed to further remove organic material. To distinguish between CeO_2_ particles and soluble CeO_2_ a semiquantitative technique was implemented using nuclear pore filters (diameter: 0.1 µm).

## Additional files

**Additional file 1: Table S1.** Fold regulation of regulated genes. List of fold regulation and standard deviation values of the regulated genes listed in Tables 2 and 3 of the manuscript. Values are shown for every dose group and time point.

**Additional file 2: Table S2.** Analyzed genes per array. List of all analyzed genes (short and long name) separated per PCR profiler array.

## Abbreviations

8-OHdG: 8-oxo-2′-deoxyguanosine
AEI cells: alveolar epithelial cells type I
AEII cells: alveolar epithelial cells type II
Alb: albumin
BAL: bronchoalveolar lavage
Birc5: baculoviral IAP repeat-containing 5
Brca1: breast cancer 1
Ccl11: C–C motif chemokine ligand 11
Ccl17: C–C motif chemokine ligand 17
Ccl2: C–C motif chemokine ligand 2
Ccl20: C–C motif chemokine ligand 20
Ccl22: C–C motif chemokine ligand 22
Ccl24: C–C motif chemokine ligand 24
Ccl3: C–C motif chemokine ligand 3
Ccl4: C–C motif chemokine ligand 4
Ccl7: C–C motif chemokine ligand 7
Ccl9: chemokine (C–C motif) ligand 9
Ccr2: chemokine (C–C motif) receptor 2
Cd40lg: CD40 ligand
COPD: chronic obstructive lung disease
Cx3cl1: chemokine (C–X3–C motif) ligand 1
Cx3cr1: C–X3–C motif chemokine receptor 1
Cxcl12: chemokine (C–X–C motif) ligand 12
Cxcl9: C–X–C motif chemokine ligand 9
Cxcr2: chemokine (C–X–C motif) receptor 2
Cxcr3: chemokine (C–X–C motif) receptor 3
Cxcr5: chemokine (C–X–C motif) receptor 5
Cyp1b1: cytochrome P450, family 1, subfamily b, polypeptide 1
Epx: eosinophil peroxidase
Exo1: exonuclease 1
Fabp4: fatty acid binding protein 4
FR: fold regulation
Gclc: glutamate-cysteine ligase, catalytic SU
Gclm: glutamate cysteine ligase, modifier SU
Gpx2: glutathione peroxidase 2
Hba1: hemoglobin alpha, adult chain 2
IFNγ: interferon gamma
Il1rn: interleukin 1 receptor antagonist
Il1α: interleukin 1 alpha
Il1β: interleukin 1 beta
Il2rb: interleukin 2 receptor, beta
Il2rg: interleukin 2 receptor, gamma
Krt1: keratin 1
LDH: lactate dehydrogenase
Lig4: ligase IV, DNA, ATP-dependent
Lpo: lactoperoxidase
LYMPH: lymphocyte
Mmp12: matrix metallopeptidase 12
MPH: macrophage
Mpo: myeloperoxidase
Msh5: mutS homolog 5
Mutyh: MutY homolog (*E. coli*)
NADPH: nicotineamide adenine dinucleotide phosphate
Ncf2: neutrophil cytosolic factor 2
Ngb: neuroglobin
Noxo1: NADPH oxidase organizer 1
OECD: Organization for Economic Co-operation and Development
Opcml: opioid binding protein/cell adhesion molecule-like
Osm: oncostatin M
Pf4: Platelet factor 4
PMN: polymorphonuclear neutrophil
ROS: reactive oxygen species
Scd1: stearoyl-Coenzyme A desaturase 1
Smug1: single-strand-selective monofunctional uracil-DNA glycosylase 1
SOD: superoxide dismutase
Spp1: secreted phosphoprotein 1
SWCNT: single walled carbon nanotubes
Tcf21: transcription factor 21
Thbs2: thrombospondin 2
Tnfsf4: tumor necrosis factor superfamily member 4
Top2a: topoisomerase (DNA) II alpha
Txnip: thioredoxin interacting protein
Xrcc2: X-ray repair complementing defective repair in Chinese hamster cells 2
γ-H2AX: histon γ-H2AX
