# Supplementary material for: Cerium oxide and barium sulfate nanoparticle inhalation affects gene expression in alveolar epithelial cells type II
Source: J Nanobiotechnology. 2018 Feb 20;16:16. doi: 10.1186/s12951-018-0343-4 (PMC5819288; doi:10.1186/s12951-018-0343-4)
Supplement: Supplementary file 2 — Additional file 2: Table S2. Analyzed genes per array. List of all analyzed genes (short and long name) separated per PCR profiler array. [file 12951_2018_343_MOESM2_ESM.docx]

Table S1 – Analyzed genes per array

| **Inflammatory Cytokines and Receptors (PARN-011Z)** | |
| --- | --- |
| Aimp1 | Aminoacyl tRNA synthetase complex-interacting multifunctional protein 1 |
| Bmp2 | Bone morphogenetic protein 2 |
| Ccl11 | Chemokine (C-C motif) ligand 11 |
| Ccl12 | Chemokine (C-C motif) ligand 12 |
| Ccl17 | Chemokine (C-C motif) ligand 17 |
| Ccl19 | Chemokine (C-C motif) ligand 19 |
| Ccl2 | Chemokine (C-C motif) ligand 2 |
| Ccl20 | Chemokine (C-C motif) ligand 20 |
| Ccl22 | Chemokine (C-C motif) ligand 22 |
| Ccl24 | Chemokine (C-C motif) ligand 24 |
| Ccl3 | Chemokine (C-C motif) ligand 3 |
| Ccl4 | Chemokine (C-C motif) ligand 4 |
| Ccl5 | Chemokine (C-C motif) ligand 5 |
| Ccl6 | Chemokine (C-C motif) ligand 6 |
| Ccl7 | Chemokine (C-C motif) ligand 7 |
| Ccl9 | Chemokine (C-C motif) ligand 9 |
| Ccr1 | Chemokine (C-C motif) receptor 1 |
| Ccr10 | Chemokine (C-C motif) receptor 10 |
| Ccr2 | Chemokine (C-C motif) receptor 2 |
| Ccr3 | Chemokine (C-C motif) receptor 3 |
| Ccr4 | Chemokine (C-C motif) receptor 4 |
| Ccr5 | Chemokine (C-C motif) receptor 5 |
| Ccr6 | Chemokine (C-C motif) receptor 6 |
| Ccr8 | Chemokine (C-C motif) receptor 8 |
| Cd40lg | CD40 ligand |
| Csf1 | Colony stimulating factor 1 (macrophage) |
| Csf2 | Colony stimulating factor 2 (granulocyte-macrophage) |
| Csf3 | Colony stimulating factor 3 (granulocyte) |
| Cx3cl1 | Chemokine (C-X3-C motif) ligand 1 |
| Cx3cr1 | Chemokine (C-X3-C motif) receptor 1 |
| Cxcl1 | Chemokine (C-X-C motif) ligand 1 (melanoma growth stimulating activity, alpha) |
| Cxcl10 | Chemokine (C-X-C motif) ligand 10 |
| Cxcl11 | Chemokine (C-X-C motif) ligand 11 |
| Cxcl12 | Chemokine (C-X-C motif) ligand 12 (stromal cell-derived factor 1) |
| Cxcl2 | Chemokine (C-X-C motif) ligand 2 |
| Cxcl5 | Chemokine (C-X-C motif) ligand 5 |
| Cxcl9 | Chemokine (C-X-C motif) ligand 9 |
| Cxcr2 | Chemokine (C-X-C motif) receptor 2 |
| Cxcr3 | Chemokine (C-X-C motif) receptor 3 |
| Cxcr5 | Chemokine (C-X-C motif) receptor 5 |
| Faslg | Fas ligand (TNF superfamily, member 6) |
| Ifng | Interferon gamma |
| Il10ra | Interleukin 10 receptor, alpha |
| Il11 | Interleukin 11 |
| Il13 | Interleukin 13 |
| Il15 | Interleukin 15 |
| Il16 | Interleukin 16 |
| Il17a | Interleukin 17A |
| Il17b | Interleukin 17B |
| Il17f | Interleukin 17F |
| Il1a | Interleukin 1 alpha |
| Il1b | Interleukin 1 beta |
| Il1r1 | Interleukin 1 receptor, type I |
| Il1rn | Interleukin 1 receptor antagonist |
| Il21 | Interleukin 21 |
| Il27 | Interleukin 27 |
| Il2rb | Interleukin 2 receptor, beta |
| Il2rg | Interleukin 2 receptor, gamma |
| Il3 | Interleukin 3 |
| Il33 | Interleukin 33 |
| Il4 | Interleukin 4 |
| Il5 | Interleukin 5 |
| Il5ra | Interleukin 5 receptor, alpha |
| Il6r | Interleukin 6 receptor |
| Il6st | Interleukin 6 signal transducer |
| Il7 | Interleukin 7 |
| Cxcr1 | Interleukin 8 receptor, alpha |
| Lta | Lymphotoxin alpha (TNF superfamily, member 1) |
| Ltb | Lymphotoxin beta (TNF superfamily, member 3) |
| Mif | Macrophage migration inhibitory factor |
| Nampt | Nicotinamide phosphoribosyltransferase |
| Osm | Oncostatin M |
| Pf4 | Platelet factor 4 |
| RGD1561905_predicted | Complement component 5 |
| Spp1 | Secreted phosphoprotein 1 |
| Tnf | Tumor necrosis factor (TNF superfamily, member 2) |
| Tnfrsf11b | Tumor necrosis factor receptor superfamily, member 11b |
| Tnfsf10 | Tumor necrosis factor (ligand) superfamily, member 10 |
| Tnfsf11 | Tumor necrosis factor (ligand) superfamily, member 11 |
| Tnfsf13 | Tumor necrosis factor (ligand) superfamily, member 13 |
| Tnfsf13b | Tumor necrosis factor (ligand) superfamily, member 13b |
| Tnfsf14 | Tumor necrosis factor (ligand) superfamily, member 14 |
| Tnfsf4 | Tumor necrosis factor (ligand) superfamily, member 4 |
| Vegfa | Vascular endothelial growth factor A |
|  |  |
| **Oxidative Stress (PARN-065Z)** | |
| Alb | Albumin |
| Als2 | Amyotrophic lateral sclerosis 2 (juvenile) homolog (human) |
| Aox1 | Aldehyde oxidase 1 |
| Apc | Adenomatous polyposis coli |
| Apoe | Apolipoprotein E |
| Cat | Catalase |
| Ccl5 | Chemokine (C-C motif) ligand 5 |
| Ccs | Copper chaperone for superoxide dismutase |
| Ctsb | Cathepsin B |
| Cyba | Cytochrome b-245, alpha polypeptide |
| Cygb | Cytoglobin |
| Dhcr24 | 24-dehydrocholesterol reductase |
| Dnm2 | Dynamin 2 |
| Duox1 | Dual oxidase 1 |
| Duox2 | Dual oxidase 2 |
| Ehd2 | EH-domain containing 2 |
| Epx | Eosinophil peroxidase |
| Ercc2 | Excision repair cross-complementing rodent repair deficiency, complementation group 2 |
| Ercc6 | Excision repair cross-complementing rodent repair deficiency, complementation group 6 |
| Fancc | Fanconi anemia, complementation group C |
| Fmo2 | Flavin containing monooxygenase 2 |
| Fth1 | Ferritin, heavy polypeptide 1 |
| Gclc | Glutamate-cysteine ligase, catalytic subunit |
| Gclm | Glutamate cysteine ligase, modifier subunit |
| Gpx1 | Glutathione peroxidase 1 |
| Gpx2 | Glutathione peroxidase 2 |
| Gpx3 | Glutathione peroxidase 3 |
| Gpx4 | Glutathione peroxidase 4 |
| Gpx5 | Glutathione peroxidase 5 |
| Gpx6 | Glutathione peroxidase 6 |
| Gpx7 | Glutathione peroxidase 7 |
| Gsr | Glutathione reductase |
| Gstk1 | Glutathione S-transferase kappa 1 |
| Gstp1 | Glutathione S-transferase pi 1 |
| Hba1 | Hemoglobin alpha, adult chain 2 |
| Hmox1 | Heme oxygenase (decycling) 1 |
| Hspa1a | Heat shock 70kD protein 1A |
| Idh1 | Isocitrate dehydrogenase 1 (NADP+), soluble |
| Ift172 | Intraflagellar transport 172 homolog (Chlamydomonas) |
| Krt1 | Keratin 1 |
| LOC367198 | Similar to Serine/threonine-protein kinase ATR (Ataxia telangiectasia and Rad3-related protein) |
| Lpo | Lactoperoxidase |
| Mb | Myoglobin |
| Mpo | Myeloperoxidase |
| Ncf1 | Neutrophil cytosolic factor 1 |
| Ncf2 | Neutrophil cytosolic factor 2 |
| Ngb | Neuroglobin |
| Nos2 | Nitric oxide synthase 2, inducible |
| Nox4 | NADPH oxidase 4 |
| Noxa1 | NADPH oxidase activator 1 |
| Noxo1 | NADPH oxidase organizer 1 |
| Nqo1 | NAD(P)H dehydrogenase, quinone 1 |
| Nudt1 | Nudix (nucleoside diphosphate linked moiety X)-type motif 1 |
| Park7 | Parkinson disease (autosomal recessive, early onset) 7 |
| Prdx1 | Peroxiredoxin 1 |
| Prdx2 | Peroxiredoxin 2 |
| Prdx3 | Peroxiredoxin 3 |
| Prdx4 | Peroxiredoxin 4 |
| Prdx5 | Peroxiredoxin 5 |
| Prdx6 | Peroxiredoxin 6 |
| Prnp | Prion protein |
| Psmb5 | Proteasome (prosome, macropain) subunit, beta type 5 |
| Ptgs1 | Prostaglandin-endoperoxide synthase 1 |
| Ptgs2 | Prostaglandin-endoperoxide synthase 2 |
| Rag2 | Recombination activating gene 2 |
| Scd1 | Stearoyl-Coenzyme A desaturase 1 |
| Vimp | Selenoprotein S |
| Sepp1 | Selenoprotein P, plasma, 1 |
| Serpinb1b | Serine (or cysteine) peptidase inhibitor, clade B, member 1b |
| Slc38a1 | Solute carrier family 38, member 1 |
| Slc38a5 | Solute carrier family 38, member 5 |
| Sod1 | Superoxide dismutase 1, soluble |
| Sod2 | Superoxide dismutase 2, mitochondrial |
| Sod3 | Superoxide dismutase 3, extracellular |
| Sqstm1 | Sequestosome 1 |
| Srxn1 | Sulfiredoxin 1 homolog (S. cerevisiae) |
| Tpo | Thyroid peroxidase |
| Txn1 | Thioredoxin 1 |
| Txnip | Thioredoxin interacting protein |
| Txnrd1 | Thioredoxin reductase 1 |
| Txnrd2 | Thioredoxin reductase 2 |
| Ucp2 | Uncoupling protein 2 (mitochondrial, proton carrier) |
| Ucp3 | Uncoupling protein 3 (mitochondrial, proton carrier) |
| Vim | Vimentin |
|  |  |
| **DNA Repair (PARN-042Z)** | |
| Alkbh2 | AlkB, alkylation repair homolog 2 (E. coli) |
| Alkbh3 | AlkB, alkylation repair homolog 3 (E. coli) |
| Apex1 | APEX nuclease (multifunctional DNA repair enzyme) 1 |
| Atm | Ataxia telangiectasia mutated homolog (human) |
| Atxn3 | Ataxin 3 |
| Blm | Bloom syndrome, RecQ helicase-like |
| Brca1 | Breast cancer 1 |
| Brca2 | Breast cancer 2 |
| Ccnh | Cyclin H |
| Cdk7 | Cyclin-dependent kinase 7 |
| Dclre1a | DNA cross-link repair 1A, PSO2 homolog (S. cerevisiae) |
| Dclre1b | DNA cross-link repair 1B, PSO2 homolog (S. cerevisiae) |
| Ddb1 | Damage-specific DNA binding protein 1 |
| Ddb2 | Damage specific DNA binding protein 2 |
| Dmc1 | DMC1 dosage suppressor of mck1 homolog, meiosis-specific homologous recombination (yeast) |
| Ercc1 | Excision repair cross-complementing rodent repair deficiency, complementation group 1 |
| Ercc2 | Excision repair cross-complementing rodent repair deficiency, complementation group 2 |
| Ercc3 | Excision repair cross-complementing rodent repair deficiency, complementation group 3 |
| Ercc4 | Excision repair cross-complementing rodent repair deficiency, complementation group 4 |
| Ercc5 | Excision repair cross-complementing rodent repair deficiency, complementation group 5 |
| Ercc6 | Excision repair cross-complementing rodent repair deficiency, complementation group 6 |
| Ercc8 | Excision repair cross-complementing rodent repair deficiency, complementation group 8 |
| Exo1 | Exonuclease 1 |
| Fen1 | Flap structure-specific endonuclease 1 |
| Gen1 | Gen homolog 1, endonuclease (Drosophila) |
| Lig1 | Ligase I, DNA, ATP-dependent |
| Lig3 | Ligase III, DNA, ATP-dependent |
| Lig4 | Ligase IV, DNA, ATP-dependent |
| LOC100360342 | MutS homolog 6 |
| Mgmt | O-6-methylguanine-DNA methyltransferase |
| Mlh1 | MutL homolog 1 (E. coli) |
| Mlh3 | MutL homolog 3 (E. coli) |
| Mpg | N-methylpurine-DNA glycosylase |
| Mre11a | MRE11 meiotic recombination 11 homolog A (S. cerevisiae) |
| Msh2 | MutS homolog 2 (E. coli) |
| Msh3 | MutS homolog 3 (E. coli) |
| Msh5 | MutS homolog 5 (E. coli) |
| Mus81 | MUS81 endonuclease homolog (S. cerevisiae) |
| Mutyh | MutY homolog (E. coli) |
| Neil1 | Nei endonuclease VIII-like 1 (E. coli) |
| Neil2 | Nei like 2 (E. coli) |
| Nhej1 | Nonhomologous end-joining factor 1 |
| Nthl1 | Nth (endonuclease III)-like 1 (E.coli) |
| Ogg1 | 8-oxoguanine DNA glycosylase |
| Parp1 | Poly (ADP-ribose) polymerase 1 |
| Parp2 | Poly (ADP-ribose) polymerase 2 |
| Parp3 | Poly (ADP-ribose) polymerase family, member 3 |
| Pms1 | Postmeiotic segregation increased 1 (S. cerevisiae) |
| Pms2 | PMS2 postmeiotic segregation increased 2 (S. cerevisiae) |
| Pnkp | Polynucleotide kinase 3'-phosphatase |
| Polb | Polymerase (DNA directed), beta |
| Pold3 | Polymerase (DNA-directed), delta 3, accessory subunit |
| Poll | Polymerase (DNA directed), lambda |
| Prkdc | Protein kinase, DNA activated, catalytic polypeptide |
| Rad18 | RAD18 homolog (S. cerevisiae) |
| Rad21 | RAD21 homolog (S. pombe) |
| Rad23a | RAD23 homolog A (S. cerevisiae) |
| Rad23b | RAD23 homolog B (S. cerevisiae) |
| Rad50 | RAD50 homolog (S. cerevisiae) |
| Rad51 | RAD51 homolog (RecA homolog, E. coli) (S. cerevisiae) |
| Rad51c | Rad51 homolog c (S. cerevisiae) |
| Rad51d | RAD51-like 3 (S. cerevisiae) |
| Rad52 | RAD52 homolog (S. cerevisiae) |
| Rad54l | RAD54 like (S. cerevisiae) |
| Rad9b | RAD9 homolog B (S. cerevisiae) |
| Rfc1 | Replication factor C (activator 1) 1 |
| Rpa1 | Replication protein A1 |
| Rpa3 | Replication protein A3 |
| Slk | STE20-like kinase (yeast) |
| Smug1 | Single-strand-selective monofunctional uracil-DNA glycosylase 1 |
| Tdg | Thymine-DNA glycosylase |
| Top3a | Topoisomerase (DNA) III alpha |
| Top3b | Topoisomerase (DNA) III beta |
| Trex1 | Three prime repair exonuclease 1 |
| Trex2 | Three prime repair exonuclease 2 |
| Ung | Uracil-DNA glycosylase |
| Xab2 | XPA binding protein 2 |
| Xpc | Xeroderma pigmentosum, complementation group C |
| Xrcc1 | X-ray repair complementing defective repair in Chinese hamster cells 1 |
| Xrcc2 | X-ray repair complementing defective repair in Chinese hamster cells 2 |
| Xrcc4 | X-ray repair complementing defective repair in Chinese hamster cells 4 |
| Xrcc5 | X-ray repair complementing defective repair in Chinese hamster cells 5 |
| Xrcc6 | X-ray repair complementing defective repair in Chinese hamster cells 6 |
| Xrcc6bp1 | XRCC6 binding protein 1 |
|  |  |
| **Apoptosis (PARN-012Z)** | |
| Abl1 | C-abl oncogene 1, receptor tyrosine kinase |
| Aifm1 | Apoptosis-inducing factor, mitochondrion-associated 1 |
| Akt1 | V-akt murine thymoma viral oncogene homolog 1 |
| Anxa5 | Annexin A5 |
| Apaf1 | Apoptotic peptidase activating factor 1 |
| Api5 | Apoptosis inhibitor 5 |
| Aven | Apoptosis, caspase activation inhibitor |
| Bad | BCL2-associated agonist of cell death |
| Bag1 | BCL2-associated athanogene |
| Bak1 | BCL2-antagonist/killer 1 |
| Bax | Bcl2-associated X protein |
| Bcl10 | B-cell CLL/lymphoma 10 |
| Bcl2 | B-cell CLL/lymphoma 2 |
| Bcl2a1 | B-cell leukemia/lymphoma 2 related protein A1d |
| Bcl2l1 | Bcl2-like 1 |
| Bcl2l11 | BCL2-like 11 (apoptosis facilitator) |
| Bcl2l2 | Bcl2-like 2 |
| Bid | BH3 interacting domain death agonist |
| Bik | BCL2-interacting killer (apoptosis-inducing) |
| Birc2 | Baculoviral IAP repeat-containing 2 |
| Birc3 | Baculoviral IAP repeat-containing 3 |
| Birc5 | Baculoviral IAP repeat-containing 5 |
| Bnip2 | BCL2/adenovirus E1B interacting protein 2 |
| Bnip3 | BCL2/adenovirus E1B interacting protein 3 |
| Bok | BCL2-related ovarian killer |
| Card10 | Caspase recruitment domain family, member 10 |
| Casp1 | Caspase 1 |
| Casp12 | Caspase 12 |
| Casp14 | Caspase 14 |
| Casp2 | Caspase 2 |
| Casp3 | Caspase 3 |
| Casp4 | Caspase 4, apoptosis-related cysteine peptidase |
| Casp6 | Caspase 6 |
| Casp7 | Caspase 7 |
| Casp8 | Caspase 8 |
| Casp8ap2 | Caspase 8 associated protein 2 |
| Casp9 | Caspase 9, apoptosis-related cysteine peptidase |
| Cd40 | CD40 molecule, TNF receptor superfamily member 5 |
| Cd40lg | CD40 ligand |
| Cflar | CASP8 and FADD-like apoptosis regulator |
| Cidea | Cell death-inducing DFFA-like effector a |
| Cideb | Cell death-inducing DFFA-like effector b |
| Cycs | Cytochrome c, somatic |
| Dad1 | Defender against cell death 1 |
| Dapk1 | Death associated protein kinase 1 |
| Dffa | DNA fragmentation factor, alpha subunit |
| Dffb | DNA fragmentation factor, beta polypeptide (caspase-activated DNase) |
| Diablo | Diablo homolog (Drosophila) |
| Fadd | Fas (TNFRSF6)-associated via death domain |
| Faim | Fas apoptotic inhibitory molecule |
| Fas | Fas (TNF receptor superfamily, member 6) |
| Faslg | Fas ligand (TNF superfamily, member 6) |
| Gadd45a | Growth arrest and DNA-damage-inducible, alpha |
| Hrk | Harakiri, BCL2 interacting protein (contains only BH3 domain) |
| Il10 | Interleukin 10 |
| Lta | Lymphotoxin alpha (TNF superfamily, member 1) |
| Ltbr | Lymphotoxin beta receptor (TNFR superfamily, member 3) |
| Mapk1 | Mitogen activated protein kinase 1 |
| Mapk8ip1 | Mitogen-activated protein kinase 8 interacting protein 1 |
| Mcl1 | Myeloid cell leukemia sequence 1 |
| Naip6 | NLR family, apoptosis inhibitory protein 6 |
| Nfkb1 | Nuclear factor of kappa light polypeptide gene enhancer in B-cells 1 |
| Nol3 | Nucleolar protein 3 (apoptosis repressor with CARD domain) |
| Polb | Polymerase (DNA directed), beta |
| Prdx2 | Peroxiredoxin 2 |
| Prlr | Prolactin receptor |
| Pycard | PYD and CARD domain containing |
| Ripk2 | Receptor-interacting serine-threonine kinase 2 |
| Sphk2 | Sphingosine kinase 2 |
| Tnf | Tumor necrosis factor (TNF superfamily, member 2) |
| Tnfrsf10b | Tumor necrosis factor receptor superfamily, member 10b |
| Tnfrsf11b | Tumor necrosis factor receptor superfamily, member 11b |
| Tnfrsf1a | Tumor necrosis factor receptor superfamily, member 1a |
| Tnfrsf1b | Tumor necrosis factor receptor superfamily, member 1b |
| Tnfsf10 | Tumor necrosis factor (ligand) superfamily, member 10 |
| Tnfsf12 | Tumor necrosis factor ligand superfamily member 12 |
| Tp53 | Tumor protein p53 |
| Tp53bp2 | Tumor protein p53 binding protein, 2 |
| Tp63 | Tumor protein p63 |
| Tp73 | Tumor protein p73 |
| Tradd | TNFRSF1A-associated via death domain |
| Traf2 | Tnf receptor-associated factor 2 |
| Traf3 | Tnf receptor-associated factor 3 |
| Xiap | X-linked inhibitor of apoptosis |
|  |  |
| **Lung Cancer (PARN-134Z)** | |
| Ager | Advanced glycosylation end product-specific receptor |
| Agr2 | Anterior gradient homolog 2 (Xenopus laevis) |
| Akt1 | V-akt murine thymoma viral oncogene homolog 1 |
| Anxa5 | Annexin A5 |
| Apba1 | Amyloid beta (A4) precursor protein-binding, family A, member 1 |
| Apc | Adenomatous polyposis coli |
| Aqp4 | Aquaporin 4 |
| Bax | Bcl2-associated X protein |
| Bcl2 | B-cell CLL/lymphoma 2 |
| Braf | V-raf murine sarcoma viral oncogene homolog B1 |
| Cadm1 | Cell adhesion molecule 1 |
| Car4 | Carbonic anhydrase 4 |
| Casp3 | Caspase 3 |
| Ccnd1 | Cyclin D1 |
| Cd36 | CD36 molecule (thrombospondin receptor) |
| Cdh1 | Cadherin 1 |
| Cdh13 | Cadherin 13 |
| Cdkn1a | Cyclin-dependent kinase inhibitor 1A |
| Cdkn1c | Cyclin-dependent kinase inhibitor 1C |
| Cdkn2a | Cyclin-dependent kinase inhibitor 2A |
| Cdkn2b | Cyclin-dependent kinase inhibitor 2B (p15, inhibits CDK4) |
| Ceacam6 | Carcinoembryonic antigen-related cell adhesion molecule 6 |
| Clic5 | Chloride intracellular channel 5 |
| Col11a1 | Collagen, type XI, alpha 1 |
| Cp | Ceruloplasmin |
| Csf3 | Colony stimulating factor 3 (granulocyte) |
| Ctnnb1 | Catenin (cadherin associated protein), beta 1 |
| Cxcl12 | Chemokine (C-X-C motif) ligand 12 (stromal cell-derived factor 1) |
| Cxcl13 | Chemokine (C-X-C motif) ligand 13 |
| Cyp1b1 | Cytochrome P450, family 1, subfamily b, polypeptide 1 |
| Dlc1 | Deleted in liver cancer 1 |
| Dlg2 | Discs, large homolog 2 (Drosophila) |
| Dusp6 | Dual specificity phosphatase 6 |
| Egfr | Epidermal growth factor receptor |
| Erbb2 | V-erb-b2 erythroblastic leukemia viral oncogene homolog 2, neuro/glioblastoma derived oncogene homolog (avian) |
| Erbb3 | V-erb-b2 erythroblastic leukemia viral oncogene homolog 3 (avian) |
| Ercc1 | Excision repair cross-complementing rodent repair deficiency, complementation group 1 |
| Fabp4 | Fatty acid binding protein 4, adipocyte |
| Fhit | Fragile histidine triad gene |
| Gpm6a | Glycoprotein m6a |
| Grem1 | Gremlin 1, cysteine knot superfamily, homolog (Xenopus laevis) |
| Hgf | Hepatocyte growth factor |
| Hif1a | Hypoxia-inducible factor 1, alpha subunit (basic helix-loop-helix transcription factor) |
| Hras | Harvey rat sarcoma virus oncogene |
| Irf4 | Interferon regulatory factor 4 |
| Kit | V-kit Hardy-Zuckerman 4 feline sarcoma viral oncogene homolog |
| Kras | V-Ki-ras2 Kirsten rat sarcoma viral oncogene homolog |
| Krt14 | Keratin 14 |
| Krt5 | Keratin 5 |
| Lck | Lymphocyte-specific protein tyrosine kinase |
| Mapk1 | Mitogen activated protein kinase 1 |
| Met | Met proto-oncogene |
| Mgmt | O-6-methylguanine-DNA methyltransferase |
| Mki67 | Marker of proliferation Ki-67 |
| Mlh1 | MutL homolog 1 (E. coli) |
| Mmp12 | Matrix metallopeptidase 12 |
| Mmp1 | Matrix metallopeptidase 1a (interstitial collagenase) |
| Mmp2 | Matrix metallopeptidase 2 |
| Mmp9 | Matrix metallopeptidase 9 |
| Mthfr | Methylenetetrahydrofolate reductase (NAD(P)H) |
| Nf1 | Neurofibromin 1 |
| Nfkb1 | Nuclear factor of kappa light polypeptide gene enhancer in B-cells 1 |
| Nkx2-1 | NK2 homeobox 1 |
| Opcml | Opioid binding protein/cell adhesion molecule-like |
| Prdm2 | PR domain containing 2, with ZNF domain |
| Ptgs2 | Prostaglandin-endoperoxide synthase 2 |
| Rassf1 | Ras association (RalGDS/AF-6) domain family member 1 |
| Rassf2 | Ras association (RalGDS/AF-6) domain family member 2 |
| Rb1 | Retinoblastoma 1 |
| Sfrp1 | Secreted frizzled-related protein 1 |
| Sftpc | Surfactant protein C |
| Sostdc1 | Sclerostin domain containing 1 |
| Spp1 | Secreted phosphoprotein 1 |
| Stat1 | Signal transducer and activator of transcription 1 |
| Stat2 | Signal transducer and activator of transcription 2 |
| Tcf21 | Transcription factor 21 |
| Tert | Telomerase reverse transcriptase |
| Tgfb1 | Transforming growth factor, beta 1 |
| Thbs2 | Thrombospondin 2 |
| Tnf | Tumor necrosis factor (TNF superfamily, member 2) |
| Top2a | Topoisomerase (DNA) II alpha |
| Tp53 | Tumor protein p53 |
| Vegfa | Vascular endothelial growth factor A |
| Wif1 | Wnt inhibitory factor 1 |
